# Functional importance of different patterns of correlation between adjacent cassette exons in human and mouse

**DOI:** 10.1186/1471-2164-9-191

**Published:** 2008-04-26

**Authors:** Tao Peng, Chenghai Xue, Jianning Bi, Tingting Li, Xiaowo Wang, Xuegong Zhang, Yanda Li

**Affiliations:** 1 MOE Key Laboratory of Bioinformatics and Bioinformatics Division, TNLIST/Department of Automation, Tsinghua University, Beijing 100084, PRoC

## Abstract

**Background:**

Alternative splicing expands transcriptome diversity and plays an important role in regulation of gene expression. Previous studies focus on the regulation of a single cassette exon, but recent experiments indicate that multiple cassette exons within a gene may interact with each other. This interaction can increase the potential to generate various transcripts and adds an extra layer of complexity to gene regulation. Several cases of exon interaction have been discovered. However, the extent to which the cassette exons coordinate with each other remains unknown.

**Results:**

Based on EST data, we employed a metric of correlation coefficients to describe the interaction between two adjacent cassette exons and then categorized these exon pairs into three different groups by their interaction (correlation) patterns. Sequence analysis demonstrates that strongly-correlated groups are more conserved and contain a higher proportion of pairs with reading frame preservation in a combinatorial manner. Multiple genome comparison further indicates that different groups of correlated pairs have different evolutionary courses: (1) The vast majority of positively-correlated pairs are old, (2) most of the weakly-correlated pairs are relatively young, and (3) negatively-correlated pairs are a mixture of old and young events.

**Conclusion:**

We performed a large-scale analysis of interactions between adjacent cassette exons. Compared with weakly-correlated pairs, the strongly-correlated pairs, including both the positively and negatively correlated ones, show more evidence that they are under delicate splicing control and tend to be functionally important. Additionally, the positively-correlated pairs bear strong resemblance to constitutive exons, which suggests that they may evolve from ancient constitutive exons, while negatively and weakly correlated pairs are more likely to contain newly emerging exons.

## Background

Alternative splicing is a post-transcriptional mechanism in which the exon sequences of primary transcripts are differently included in mature RNAs. It expands proteomic diversity and regulates developmental or tissue-specific processes by generating multiple different transcripts from a single gene [1-3]. Recent comparison of data from transcript sequencing and microarray profiling indicates that alternative splicing is more frequent in higher eukaryotes. Independent studies estimate that 40–60% of human genes undergo alternative splicing [4-7]. Mutations that disrupt splicing and/or alternative splicing are reported to be an important source of many human diseases [8-11]. Thus, many efforts have been invested in understanding the regulation of alternative splicing [12-17].

Alternative splicing is a complex phenomenon. There are four basic forms of alternative splicing. (1) Exon skipping (also known as cassette exon), in which the alternative exon is either included or skipped in the mature mRNA transcript as a whole, is the most common alternative splicing event in mammal. The next two types are (2) alternative donor splice site and (3) alternative acceptor splice site. These two types of alternative exons are flanked on one side by a constitutive splice site and on the other side by several competing alternative sites. Finally, (4) intron retention is an abundant form of alternative splicing in plants but relatively rare in mammals [18]. Combinations of the simple forms come together to make up complex types of alternative splicing, which account for 33% of all the conserved events between human and mouse [5,19,20]. Furthermore, there are many genes containing more than one region that is alternatively spliced. The possible coordination between different alternative regions composes an extra layer of complexity in alternative splicing regulation. Some recent studies have identified cases in which different alternative exons belonging to the same gene appear to be coordinated [21-23]. Fededa et al. constructed a minigene carrying two alternative EDI regions in tandem. Mutations that change the alternative splicing of the upstream EDI deeply affect the splicing pattern of the downstream one [21]. The CACNAIG gene is another example. Emerick et al. analyzed hundreds of full-length cDNA sequences of this gene and found evidence for both pair-wise and high-order correlations between different alternative exons [23].

These studies highlight the importance of investigating to what extent the coordination between alternative exons occurs in a large number of genes. Studying the coordination patterns between alternative exons and their functional importance on a genome-wide scale can provide valuable information for our understanding of alternative splicing. This information will allow researchers to further investigate individual, or 'modular', alternative splicing events in the light of the cooperative effects between alternative splicing events and gain a deeper insight into the molecular functions and regulatory mechanisms of gene control [23,24]. The alternative exon coordination information will also help researchers to eliminate those transcript forms which are unlikely to appear when considering all the possible combinations of multiple alternative regions. Narrowing down the whole transcript-form space can greatly enhance efforts to catalogue and construct a full length transcriptome [22].

**Figure 1:**
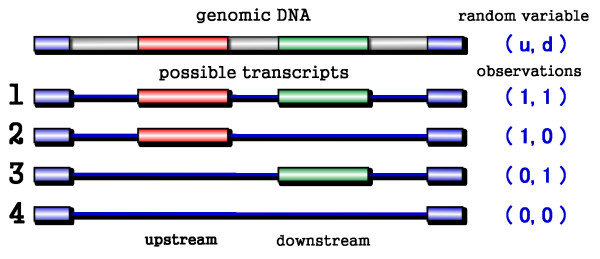
**The correlation coefficients between adjacent alternative exons**. The DNA loci and all possible transcripts for two adjacent alternative exons are shown in this graph. Lengths are not to scale. Red and green boxes depict upstream and downstream alternative exons, respectively. Blue boxes are flanking constitutive exons. The random variable u represents the upstream exon and *d *represents the downstream one. On the right of each transcript are corresponding observed values of (*u*, *d*). Given all the ESTs covering these two exons, the correlation between exons can be calculated as the correlation between the two random variables (see text for details).

To study the coordination between alternative exons, we performed a large scale analysis of the correlation (coordination) between the two exons in an adjacent cassette exon pair. An adjacent cassette exon pair consists of two exons which are both cassette exons and are adjacent in the final transcript (Figure 1). We employed a correlation coefficient to measure the interaction between the two exons in such a pair and observed that the exons show various interaction patterns. The adjacent pairs were then categorized into three groups according to their correlation coefficients. We found that strongly-correlated pairs are more conserved and that a higher proportion of strongly-correlated pairs preserve the protein reading frame. Both of these observations are evidence that these pairs are under natural selection and tend to be functionally important. Multiple genome comparison further indicates that different correlated pairs have different evolutionary courses: the vast majority of positively-correlated exon pairs are very old, while most of the weakly-correlated pairs are relatively young. The negatively-correlated group contains both many young and old events. All these observations are consistent in both human and mouse. Our analysis provides primary results about the interactions between adjacent cassette exons on a genome-wide scale.

## Results

Adjacent cassette exons are a pair of cassette exons which are adjacent in the final transcript (Figure 1). Our study focused on adjacent cassette exons because they are the simplest form of possible exon interaction and these events could be covered by sufficient ESTs. Of the two exons in an adjacent cassette exon pair, the one in the 5' upstream region is called the upstream exon and the other the downstream exon. Consider a case of an adjacent cassette exon pair, let random variable *u *represent the inclusion/exclusion status of the upstream exon in a mature transcript and let *d *represent the status of the downstream exon (Figure 1). An EST/cDNA sequence *i *spanning over these two exons will correspond to an observation of random variables (*u*_*i*_,*d*_*i*_). There are 4 possible value combinations of the pair *(u, d)*:

1) *(1, 0)*, the upstream exon is included while the downstream exon is excluded;

2) *(0, 1)*, the upstream exon is excluded but the downstream exon is included;

3) *(1, 1)*, both are included;

4) *(0, 0)*, both are excluded.

The interaction between random variable pair *(u, d) *can represent the interaction between the adjacent cassette exons. Correlation coefficient was taken as the metric to estimate this interaction (see Methods).

To obtain our dataset we extracted all of the adjacent cassette exons deposited in the Altsplice database of the ASD project ([25], see Methods), resulting in 4326 adjacent cassette exon pairs from 2451 genes in human and 1905 adjacent cassette exon pairs from 923 genes in mouse (Information for all pairs are listed in additional file 2). To obtain a relatively reliable value of correlation coefficient, we took into account only those pairs with more than 10 EST sequences covering the alternatively spliced region Of all the pairs, most (3003 in human and 1314 in mouse) passed this filter. A large portion of them had substantially higher EST coverage: the median number of ESTs excluding the filtered pairs is 37 and 31 for human and mouse, respectively. (The distribution of EST evidence number per exon pair is shown in supplementary Figure S1.)

### Different patterns of correlation between adjacent cassette exons

**Figure 2:**
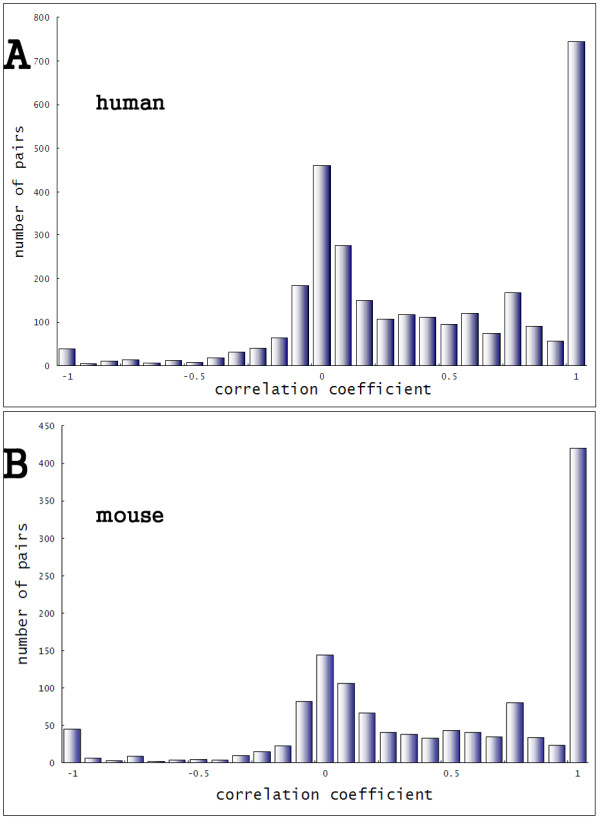
**Distribution of correlation coefficients of alternative exon pairs in human and mouse**. (A) human and (B) mouse. The x-axis is the correlation coefficient (See text) and y-axis is the number of pairs in each bin. These two histograms are very consistent regardless of the data amount. Generally, the two exons in an adjacent alternative exon pair are more likely to be positively correlated (*r > 0*). We can further identify two major and one minor peaks at 1, 0 and -1. A coefficient of -1 means the two alternative exons in a pair are strictly mutually exclusive, while 0 and 1 indicate independent and linked inclusion/exclusion, respectively. Thus, we divided the adjacent pairs into three groups: mutually exclusive (*r *≤ -0.7, ME for short), independent (-0.2 ≤ *r *≤ 0.2, IND for short) and linked (0.7 ≤ *r*, LNK for short). These three groups show different regulatory properties and functional importance.

To explore the interaction pattern of adjacent cassette exons, we first calculated the correlation coefficient of all the exon pairs in human and drew its distribution (Figure 2A). On the distribution, it can be seen that there are more positively correlated exon pairs (2350 with *r *> 0) than negatively correlated pairs (648, with *r *< 0). This asymmetry is consistent with a recent microarray study [24], in which it is reported that more cassette exon pairs positively correlated in a tissue-specific manner.

The distribution in Figure 2A shows strong multimodality. Two major and one minor peaks can be identified: 1) many of the coefficients center at zero; 2) a portion of comparable amount fall into a narrow bin near 1, forming a very sharp peak; 3) the minor peak is at -1, with less data relative to the other two peaks. Though the total amount of data is less than in human, the correlation pattern in mouse is very similar (Figure 2B). There are also more positively correlated pairs than negatively correlated ones. Three peaks can also be identified at the same places in the mouse distribution (Figure 2B). The small peak at -1 is clearer, compared with its counterpart in human. The multimodality reflects that the two exons in pairs interact with each other in different ways. We may expect different regulatory properties and functional importance among the different groups formed by these peaks.

Based on this rationale, we selected the adjacent cassette exon pairs around each of the three peaks and constructed three different groups. The first is the mutually exclusive group (*r *= *-0.7*, ME for short). The second is the independent group (*-0.2 *= *r *= *0.2*, IND for short) and the third is the linked group (*0.7 *= *r*, LNK for short). To eliminate possible noise, our analysis did not include the "gray" regions (*-0.7< r < -0.2 *and *0.2 < r < 0.7*). The numbers of human adjacent cassette exon pairs in ME, IND and LNK group are 60, 1137 and 957, respectively (Table 2). The same grouping was performed on mouse and the numbers of mouse pairs in ME, IND and LNK are 60, 424 and 507, respectively (Table 2). In the following text, we also refer ME and LNK groups as the strongly-correlated groups, and IND as the weakly-correlated group.

**Table 1:** Sequence lengths among different groups

	**Human**			**mouse**		
	
	**ME**	**IND**	**LNK**	**ME**	**IND**	**LNK**
**Upstream intron**	1293	1787	2326	1077	1968	2069
**Upstream exon**	115.5	108	113	108	104	113
**Intermediate intron**	551	1941	731	293	2234	899
**Downstream exon**	120.5	113	115	106.5	106	107
**Downstream intron**	1625.5	1696	1717	1713.5	2207	1695

**Table 2:** Frame preservation among different groups

		**# of pairs**	**# in CDS**	# of Frame preservation*			
				
				**Upstream**	**Downstream**	**Difference**	**Sum**
**Human**	**ME**	60	51 (85%)	24 (47.1%)	17 (33.3%)	36 (70.6%)	19 (37.3%)
	**IND**	1137	810 (71.2%)	349 (43.1%)	338 (41.7%)	306 (37.8%)	294 (36.3%)
	**LNK**	957	834 (87.1%)	275 (33.0%)	300 (36.0%)	292 (35.0%)	357 (42.8%)
**mouse**	**ME**	60	51 (85%)	19 (37.3%)	24 (47.1%)	39 (76.5%)	20 (39.2%)
	**IND**	424	244 (57.5%)	101 (41.4%)	113 (46.3%)	92 (37.7%)	91 (37.3%)
	**LNK**	507	424 (83.6%)	173 (40.8%)	173 (40.8%)	161 (38.0%)	190 (44.8%)

* The 'upstream' and 'downstream' indicate the numbers of frame-preserving upstream and downstream exons, respectively. The 'difference' indicates the number of pairs in which the length difference of the two exons is an exact multiple of 3 nt, while the 'sum' means the number of pairs in which the exon length sum is an exact multiple of 3 nt. The percentage is relative to '# in CDS' column. See text for detail.

Mutually exclusive alternative splicing events have long been noticed. Many researchers have labeled it as one of the five basic alternative splicing types (together with exon skipping, alternative acceptor, alternative donor and intron retention) [2]. The definition of mutual exclusion reflects people's concern that alternative splicing may be regulated by different cellular conditions. For example, an alternative splicing event with transcript form 2 and transcript form 3 from Figure 1 will be reported as a mutually exclusive event in the ASAP [26] and ASD [25] annotations, because these two transcripts may be the splicing product of two different cellular conditions (for instance, two tissue types). However, from a mathematical point of view, mutual exclusivity is not a property of a certain transcript but instead describes the relationship between differently spliced transcripts, which makes it difficult to give an accurate definition. When considering all the possible transcript forms, our data suggests that the strictly "mutually exclusive" exons count as only a small portion of all the pairs.

The correlation coefficient estimates the correlation between two binary variables (inclusion/exclusion states of upstream and downstream exons). The correlation assumes that both variables are normally distributed and that the bivariate distribution is multivariate normal, which is not satisfied in our data. The small value of EST counts further makes such estimation not very accurate. To eliminate the possibility that the distribution pattern of correlation coefficients is an artifact introduced by the relatively small value of EST counts, we performed a re-sampling procedure and a permutation procedure. The results show that the two observations in the distribution: 1) more positively-correlated pairs and 2) multimodality, are very unlikely to be random results (P-value < 1e-5. See supplementary material for the details of these results and methods from the two procedures). We also checked the correlation between the two exons by using Fisher exact test (as in [21], See supplementary for the calculation and discussion of the statistical test). The P-values of pairs are consistent with the Pearson correlation coefficients: A pair with small P-value tends to be strongly (positive or negative) correlated. The P-values for each pair, together with the FDR (False Discovery Rate) correction, are listed in additional file 2 for readers who concern high confident pairs.

### Splice site strength and exon/intron length associated with different correlations

The three groups were examined to see if there are any differences in the sequence properties. The strength of the splice site, which is important in the regulation of splicing [27], was first investigated. In general, the splice sites in cassette pairs, including ME, IND and LNK, are a little bit weaker than in constitutive exons, which is consistent with previous results that constitutive splice sites are stronger than alternative ones [28,29]. However, we did not observe meaningful differences among the ME, IND and LNK groups (see Table S1).

Next we examined the exon and intron lengths in human (Table 1). The exons of different groups are of similar length, while the intermediate introns (the intron between upstream and downstream exons) in the strongly-correlated groups (ME and LNK) are much shorter than in the weakly-correlated group (IND) (P-value roughly equals to zero, Wilcoxon test). The median lengths of intermediate introns for ME, IND and LNK are 551, 1935, and 731 nt, respectively. This result reflects that a short intermediate intron plays a role in the delicate control of strongly-correlated pairs. The intermediate introns in ME are extremely short. This may reflect the fact that steric interference is an important mechanism resulting in mutually exclusive splicing [30]. If two adjacent exons get too close to each other, the intron between them cannot be efficiently spliced out by the spliceosome. Thus, no transcript, including both exons, will be generated. In the ME group, 18 out of 60 transcripts in human and 21 out of 60 transcripts in mouse have an intermediate intron of less than 100 nt. Possibly steric interference accounts for the mutually exclusivity of these pairs.

The lengths of upstream introns (the intron in the 5' flanking of the upstream exon) are also significantly different between groups (LNK > IND > ME, P-value < 0.005). But the downstream introns (intron flanking the 3' side of the downstream exon) are almost the same length. Previous studies report that the upstream intron length affects alternative splicing [31]. We observed its impact in our analysis of cassette exon pairs. These observations were also found in the mouse data (Table 1). In the LNK group, the two exons in a pair are included/excluded simultaneously, behaving like an individual exon. The short intermediate intron and long upstream intron may be required for this synchronization.

### Strongly correlated pairs show increased selection pressure for combinatorial reading-frame preservation

An important aspect of studying alternative splicing is to find which splice events are functionally significant [32]. We investigated whether the different exon correlation patterns are associated with different functional importance. Reading-frame preservation has long been adopted as a criterion to find possible functional cassette exons [17,28]. If the length of a cassette exon is a multiple of 3 nt, the inclusion or skipping of this exon will not alter the protein reading frame of subsequent exons. Thus, a functional exon skipping event tends to be frame-preserving.

We first checked those adjacent pairs that have both exons in CDS with the annotations from UCSC knownGene [33] (See Methods). The proportions of pairs with both exons in CDS are 85%, 71.2% and 87.1% for ME, IND and LNK groups, respectively (Table 2). Strongly-correlated pairs are more likely to be located in CDS (P-value < 2.2e-16, IND vs. LNK and ME, Fisher exact test). It has been reported that exons which are more functionally important (evolutionary old) have a higher proportion of exons in CDS compared with newly emerging exons [34].

Next we checked the proportion of these possible protein-coding exons which preserve the reading frame. The results show that, in all groups, the fractions of exons whose lengths are multiples of 3 nt are just above one third, roughly equal to the proportion expected by chance (Table 2). However, if the correlation of the adjacent exons is functionally important it may not be necessary that the lengths of both the upstream and downstream exons be exact multiples of 3 nt. For example, the skipping of a 50 nt exon will cause a shift of -2 in the reading frame for subsequent exons in the final transcript. But, if the next exon of 53 nt is included, as in a ME pair, the subsequent exons will get another +2 shift. In this case, the net shift is 0 for subsequent exons and frame disruption is limited to a local region. The overall reading frame remains intact. Thus, the transcript will preserve the reading frame when the length difference of the two exons is an exact multiple of 3 nt in the ME group. Similarly, in the LNK group, a length sum could preserve the protein ORF. We did observe significant signals in exon length combinations among different groups, both in human and mouse (Table 2). When considering exon length difference, the frame-preserving proportion of ME increases to 70.6% in human and 76.5% in mouse, while it remains roughly one third in the LNK and IND groups. This difference in proportions is significant (P-value < 1.3e-6 for human, P-value < 8.4e-8 for mouse. ME vs. non-ME, Fisher-exact test). We also observed in the LNK group an increased proportion of the exon length sum being frame-preserving (42.8% in human and 44.8% in mouse) compared to ME and IND. This increase is partially significant (P-value < 7.3e-3 for human, < 0.055 for mouse, LNK vs. non-LNK, fisher-exact test). The relatively weak signal in the LNK group may be partly attributed to the different exon inclusion levels (defined as the fraction of transcripts that include this pair among all transcripts) among groups (See following).

The LNK and ME pairs show a significant increase in the reading-frame preservation in a combinatorial manner. These observations may be the result of natural selection and suggest a functional importance for exon correlation.

### Strongly correlated cassette exons are more conserved

**Figure 3:**
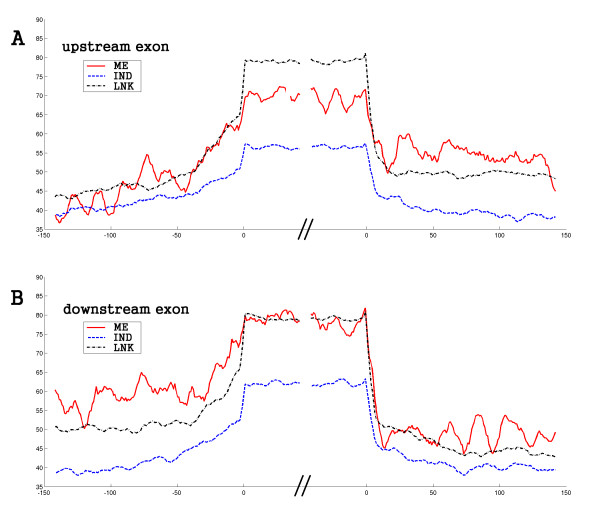
**Sequence conservation of different groups in human**. The graphs show the conservation curve of human adjacent alternative exons around the donor and acceptor sites, based on the alignment between human and mouse (human-referenced, see methods). The x-axis indicates the positions of nucleotides and the y-axis is the average percent identity in a 9-base sliding window. The range of x is 50 nt into the exon and 150 nt into the intron. Subfigure (A) and (B) are for upstream and downstream exons, respectively. Exons in LNK are most conserved, and ME exons are more conserved than IND. The flanking introns in the strongly-correlated groups (ME and LNK), especially the intermediate intron (the intron between upstream and downstream exons), are more conserved than in IND. Of the ME and LNK groups, the intermediate introns in ME show even more conservation. This observation indicates the strongly-correlated groups may be under more delicate regulatory control. Another observation is that the downstream exons are more conserved than upstream ones, especially in the ME group. This reflects the different age of upstream and downstream alternative exons (See text).

Several independent studies indicate that conserved splice variants, relative to species-specific splice variants, are more likely to play important roles [17,35]. We examined whether the three distinct groups characterized by exon correlation show differences in sequence conservation. The human-mouse genome alignment was downloaded from UCSC and mouse DNA segments were extracted according to the coordinates of the human exon (see methods). The average percent identity for alignment of the upstream exon, as well as flanking introns, was calculated (Figure 3A), and so was the downstream exon (Figure 3B). From the figure of the upstream exon, we found that exons in LNK are the most conserved, followed by exons in ME, and then the exons in IND. The LNK exons are as conserved as constitutive exons and the conservation level of the ME exon is roughly equal to the individual cassette exons in exon skipping events with only one exon skipped (data not shown). The strongly-correlated pairs, ME and LNK, are more conserved than IND pairs, suggesting exon interaction regulation may be the result of selection in evolution. The situation for downstream exons is slightly different. Compared with the upstream exon, the downstream exon in a ME pair is more conserved. Its conservation reaches the level of the LNK exons (Figure 3B).

The flanking introns in the ME and LNK groups are also more conserved than those in IND (Figure 3). The intron conservation level in strongly-correlated groups is even higher than the level of introns flanking constitutive exons (data not shown). This is similar to findings from previous studies which reported that the introns flanking conserved cassette exons are on average significantly more conserved than both introns flanking constitutive and species-specific cassette exons [17,36]. This would indicate the regulatory role of these introns. The high intron conservation observed here again supports the possibility that exon interaction regulation is associated with functional importance. Intriguingly, in the strongly-correlated pairs, the intermediate intron (between two exons) is more conserved than the upstream and downstream introns (Figure 3). This conservation difference is more significant in the ME group.

**Figure 4:**
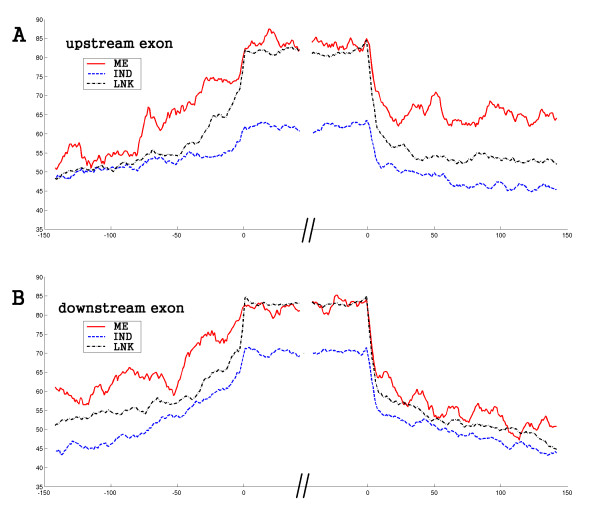
**Sequence conservation of different groups in mouse**. The graphs show the conservation curve of mouse adjacent alternative exons around the donor and acceptor sites, based on the alignment between mouse and human (mouse-referenced, see methods). The x- and y-axis are the same as Figure 3. Results in the mouse are consistent with what was seen in the human: the strongly-correlated groups are more conserved than the weakly-correlated group, in both exon and flanking introns; the intermediate introns in the strongly-correlated groups are even more conserved. The conservation difference is also observed between the upstream and downstream exons. Compared with Figure 3, the difference between the upstream and downstream exons is less pronounces for the ME group, but it's more clear in the IND group.

The canonical splicing signals, branch site and poly-pyrimidine tract (PPT), overlap with the intron region showing conservation difference. To eliminate the possibility that the length of the branch site and PPT account for the observed conservation difference, we predicted the distance from branch site to splice site and the length of PPT in the flanking introns. The results show that the distance from the branch site to the splice site and the length of the PPT are similar between the different groups (Table S2). Thus, the canonical splicing signals are not a main cause of this observation. It seems that the intermediate intron in the strongly-correlated group is short and possibly contains a higher density of splicing control elements around the acceptor and donor sites. All these results, including the conservation differences of exons and flanking introns among groups, were also observed in the mouse data (Figure 4).

We observed a conservation difference between the groups on a sequence/nucleotide level. So, we also looked at how many adjacent cassette pairs are conserved as alternative splicing events between human and mouse, that is, how many cassette pairs in one species are still cassette pairs in the other. The results in Table 3 show that the proportion of conserved cassette-pairs in the strongly-correlated groups, especially ME, are significantly higher than the IND group (P < 3.5e-16, ME vs. IND, P < 1.1e-4, LNK vs. IND for human; P < 6.9e-9 ME vs. IND, P < 1.4e-4 for mouse). This is consistent with our observations on the sequence/nucleotide level. Compared with the proportion of conserved single cassette exons (10%~20% between human and mouse [17,32]), the proportion of conserved pairs in the IND group is quite low: 1.4% in human and 4.3% in mouse (Table 3). However, we would need many more ESTs to capture a cassette pair event than a single cassette exon. So the proportions in IND are reasonable considering many cassette-pairs may be missed due to a limited amount of EST data.

**Table 3:** Evolutionary course of pairs among different groups

		**conserved**	**duplication**	**repeats**	Exon age difference *		
					
					**Upstream young**	**equal**	**Upstream old**
**human mouse**	**ME**	18 (30.0%)	24 (40.0%)	8 (13.3%)	17	33	10
	**IND**	16 (1.41%)	29 (2.55%)	356 (31.3%)	419	460	258
	**LNK**	40 (4.18%)	25 (2.61%)	55 (5.74%)	125	741	91
	**ME**	18 (30.0%)	25 (41.7%)	6 (10.0%)	18	31	11
	**IND**	18 (4.25%)	6 (1.42%)	100 (23.6%)	154	195	75
	**LNK**	56 (10.1%)	11 (2.17%)	12 (2.37%)	74	389	44

* Each exon in the adjacent pairs is assigned an age label: "young", "middle" and "old". The "Upstream young" means the number of pair in which the upstream exon is younger than the downstream one. The "equal" means the number of pair in which the two exons are of equal age. The "Upstream old" means the number of rest pair with an older upstream exon.

### The majority of LNK cassette exons are major-form exons

**Figure 5:**
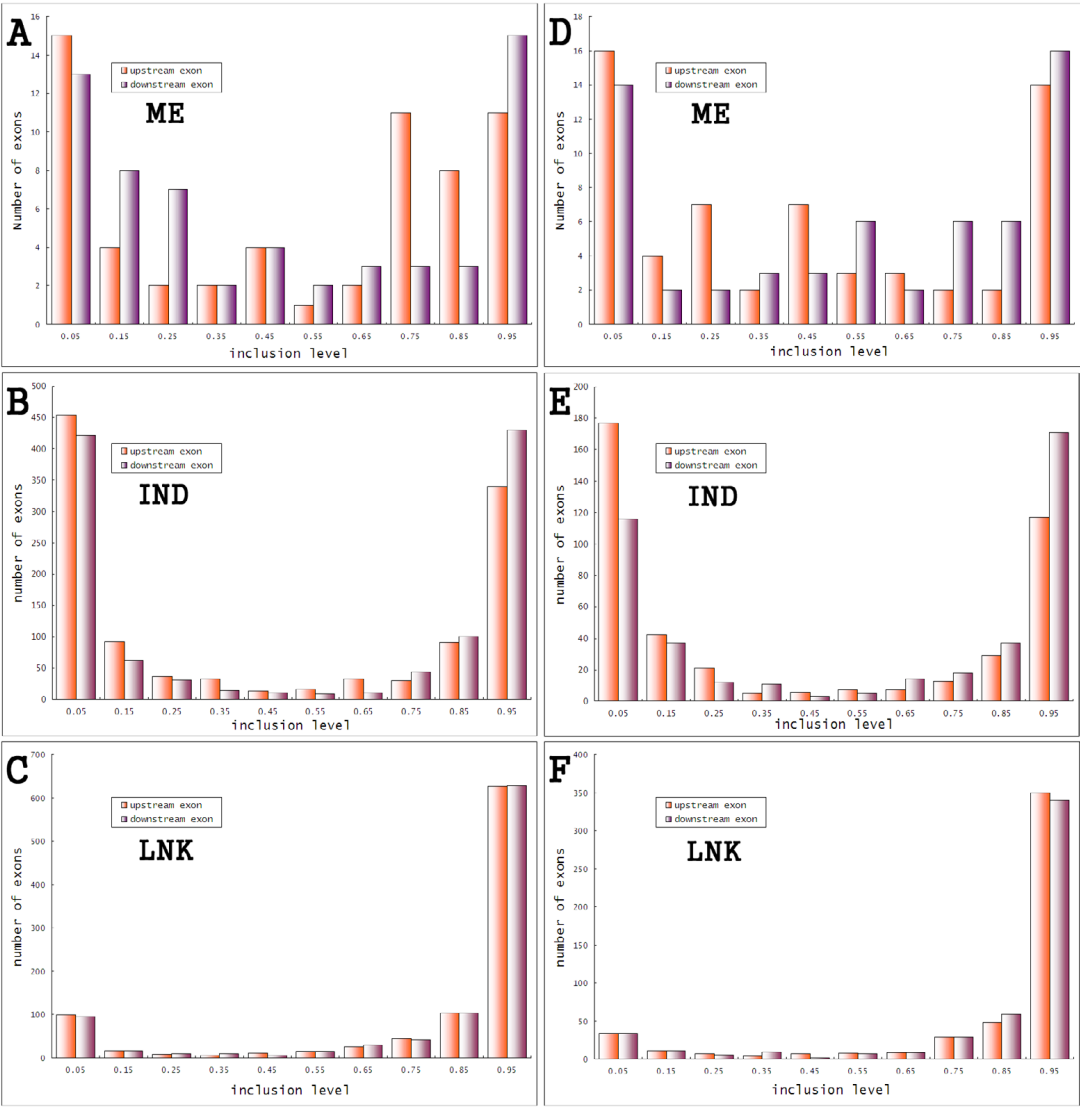
**Inclusion level of different groups**. For each cassette exon, we counted the number of ESTs in which the exon is present (*Np*) or absent (*Na*), then calculated the inclusion level as *Np*/(*Np*+*Na*). The left column is for human and the right is for mouse. The frequencies of inclusion levels in ME, IND and LNK are quite different. Exons in LNK tend to be included often, which indicates that LNK transcripts with exon are the dominating form. Exons in IND are included at either a very high or very low level. Inclusion levels in ME are similar to IND, but there are more cases in which the transcripts with or without the cassette exon are express at similar levels. These results are observed in both mouse and human.

We used the inclusion level, defined as fraction of the ESTs spanning this region and including this exon rather than skipping it, to analyze our adjacent cassette exon pairs. Several studies employ this metric as an important indicator of functional importance and evolutionary history [15,34]. The major-form splice variant (transcript with a high inclusion level) is usually conserved, while the minor form, which is found in relative low abundance, is frequently associated with recent exon creation/loss. We analyzed the inclusion level of exons in each group. The distributions of inclusion level in different groups showed distinct patterns, which is consistent in both human and mouse (Figure 5). The cassette exons in IND are either included at a very high or a very low level (Figure 5A). There are few cases in which the two splice variants, with or without the cassette exon, express in comparable abundance, i.e. 50% to 50%. This distribution is similar to the inclusion level distribution of individual cassette exons (Figure S3). This result is also consistent with previous reports that state that usually one splice variant of the possible two is dominating and functionally important [15,37]. The distribution of ME is similar to that of IND, but there are more exons with a moderate inclusion level (Figure 5B). A moderate inclusion level means the two splice variants are expressed at comparable level. Since the ME pair is regulated, it is more likely that both of the two variants in a ME event play important functional roles and thus are expressed at a similar level.

The results for the LNK group are entirely different: the distribution is heavily skewed toward 1. Most exons in LNK are major-form exons with very high inclusion level (Figure 5C). This is consistent with the high sequence conservation in LNK and may also partially explain the low protein frame preserving pressure observed in our study. Of the two different transcripts, with or without LNK exon pair, those transcripts in which the LNK pair is skipped express in low abundance. Though skipping of the LNK pair may break the reading frame, the low abundance of this defective transcript will probably not cause severe loss of overall product activity. It has been reported that major form exons exhibit low selection pressure in frame preservation [38].

Related to our observation that LNK pairs are usually have a high inclusion level, Plass et al. showed that the exonic structure is more conserved at higher inclusion levels, and that this correlates with the sequence conservation of the alternative exons [39]. From this point of view, the conserved LNK pairs can be thought of as transcript fragments with conserved exonic structure and with high inclusion level. Thus, the previous work and our analysis came to the same conclusions from two different aspects.

### Evolutionary course of adjacent cassette exon pairs

Our study indicates that the strongly-correlated groups (ME and LNK) tend to be more conserved and functionally important. So, we will investigate the origin and evolutionary course of different interaction patterns. Exon duplication has long been known as a source of mutually exclusive splicing [40,41]. We detected 78 duplications in all 2154 adjacent pairs in human (with the same criteria as [41], see methods). The exon duplications are significantly enriched in ME (Table 3): 40.0% of ME pairs arise from exon duplication, while only 2.55% of IND and 2.61% of LNK are from this origin (P < 2.2e-6, ME vs. non-ME). This enrichment is also observed in the mouse data (P < 2.2e-6, ME vs. non-ME). It has been hypothesized that most duplicated exons are mutually exclusively spliced [41]. But, with our more strict criteria, mutually exclusive pairs count only a very small proportion of all the adjacent cassette exon pairs. Though a much higher portion of ME pairs arise from exon duplication, most pairs originating from duplication interact in an independent or linked manner.

Another important source of cassette exons in vertebrates is the exonization of interspersed repeat sequences [42,43]. To systematically examine whether these repeat elements contribute to our correlated pairs, we employed RepeatMasker to identify the exons originating from repeats (See methods). Strikingly, in human, only 5.74% of the pairs in the LNK group overlap with repeat elements, while the numbers for ME and IND groups are 13.3% and 31.3%, respectively (Table 3). Most of the repeat elements are Alu, which is a primate-specific SINE element. This finding indicates many ME and IND pairs emerged in recent genome evolution and there are few recent emergences of exons in the LNK group (P < 2.2e-16, LNK vs. non-LNK). The same trend can be observed in the mouse (Table 3).

To further investigate the evolutionary course of exon interactions, we took a multiple genome comparison strategy similar to that of Zhang et al. [34]. The age of an exon is determined by its conservation in the most divergent organism. The rationale, for example, is that a human exon whose ortholog is present in a given organism must have been "born" before the divergence between human and that organism. All exons are divided into three groups: young, middle, and old (the evolutionary scale is limited to vertebrates, see methods). The age of an exon pair is then treated as the age of the younger exon in this pair. Zhang et al. [34] reported that the younger exons are more likely to be alternatively spliced and have a low inclusion level. It has also been reported that the younger exons are more likely to originate from repeat elements and be located in the UTR region. These results are consistent with our observation from our pair analysis (see following).

**Figure 6:**
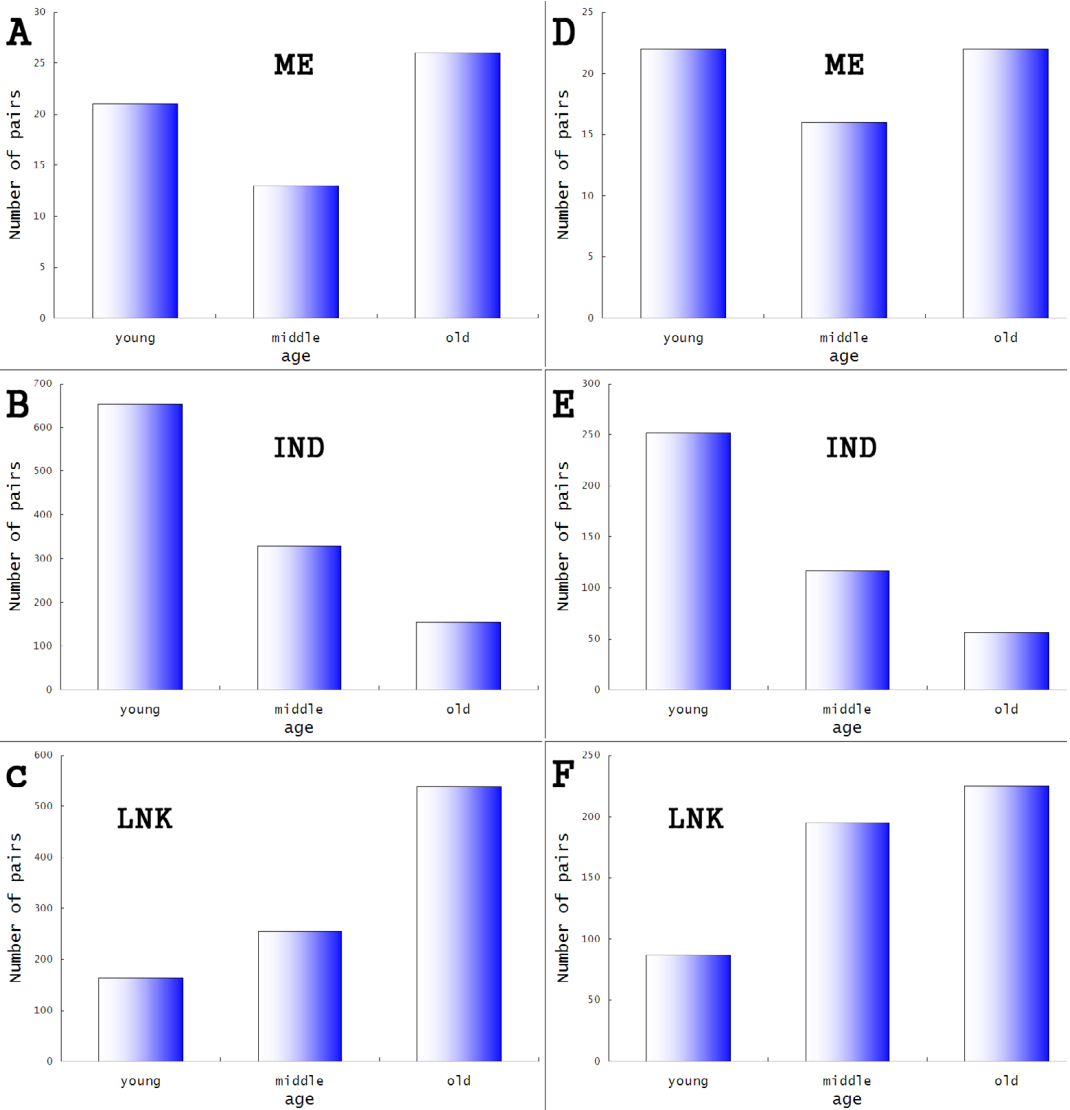
**Age of adjacent pairs in different groups**. The adjacent alternative pairs are assigned one of the three labels: "young", "middle" and "old", based on the multiple genome alignment (See text and method). The figures show the distributions of age in different groups. Most LNK pairs are old: the younger the age, the fewer pairs. IND shows the opposite trend: The older, the fewer. ME group is a mixture of LNK and IND. The numbers of young and old pairs in ME are roughly equal. These distributions indicate possible different evolutionary courses of the three groups. All the distributions are consistent in human and mouse. However, the label assignment procedures are different in human and mouse, so the amount of the data of each label can not be compared directly.

We observed distinct differences in the distribution of the pair age between the LNK, ME and IND groups (Figure 6). Most pairs in the LNK group are old. There is a relatively low proportion of LNK pairs emerging in recent genome evolution. The age distribution in the IND group is reversed compared with the LNK group (Figure 6). Most pairs in IND are very young, which is consistent with our analysis about exons overlapping from repeat elements. The situation in ME is more interesting. Of the 60 human ME pairs, 26 are evolutionary old. All the 26 pairs are located in CDS region and 24 pairs of 26 preserve the protein reading frame (the upstream and downstream exon length difference are exact multiples of 3 nt.). There are also many newly born ME exon pairs. Of the 21 young pairs, there are only seven which are both located in CDS and preserve the protein reading frame. The origins of the old and young ME pairs are also different. 19 of 26 old pairs show significantly high similarity between two exons in a pair, while no exon duplication event can be detected in the 21 young pairs. Of 8 pairs arising from repeat elements in ME (Table 3), six are young pairs and none is old. Results are similar for the mouse data.

Compared with the ME and IND groups, exons in the LNK group are old. The age difference between two exons in a pair confirms this observation. In human, the proportion of pairs from LNK in which two exons are of different age is 22.6% (216/957), while the proportions for ME and IND group are 45.0% (27/60) and 59.5% (677/1137), respectively. In mouse, the numbers are 48.3% (29/60), 54.0% (229/424), and 23.3% (118/507) for ME, IND and LNK, respectively (Table 3). LNK pairs tend to be ancient events and the two exons in a LNK pair are uniformly old, while exon ages in an ME/IND are more likely to be different and many exons emerged in recent years (all p-value < 2.5e-4, LNK vs. ME/IND).

The two exon ages in a pair are often different. An interesting observation is that the upstream exon is usually younger than downstream one. The human exon conservation curves (Figure 3), in which the downstream exons are more conserved, especially for ME, are evidence for this idea. In Table 3, there are more upstream-young pairs, defined as pairs of which upstream exon is younger than downstream one, than upstream-old pairs in each group. We observed this in both human and mouse. A permutation (see supplementary, Fig S5) confirmed the upstream-downstream asymmetry in the LNK and IND groups (P < 1e-4). The asymmetry is not significant in ME, probably due to the limited amount of data. The fact that the upstream exon is younger than the downstream exon may be because a change in the upstream exon is more likely to affect the downstream one. When a new cassette exon is generated, by mutations or other genetic variation, this change may also affect the splicing of the subsequent exon. These two exons thus result in an adjacent cassette pair. There is an experiment reporting that some exon interactions are polar: mutations on the upstream exons deeply affect the splicing of the downstream exon, while the similar mutations on the downstream exon have no effect on the upstream exon [21]. However, more systematic studies are needed to validate and explain the difference between upstream and downstream exons.

## Discussion

Currently, most studies concerning regulation of alternative splicing focus on a single alternative exon. By using EST data covering adjacent cassette exon pairs in human and mouse, we used the correlation coefficient to describe the interaction between the adjacent cassette exons and demonstrated that these pairs showed various correlation patterns. Those pairs are then categorized into three groups according to the correlation coefficient: ME (mutually exclusive pairs), IND (independent pairs) and LNK (linked pairs). The three groups show little difference in exon length and splice site strength. But the strongly-correlated pairs (ME and LNK) have a much shorter intermediate intron (the intron between upstream and downstream exons) than weakly-correlated pairs (IND). Comparison between human and mouse illustrates that the sequences in ME and LNK are more conserved, in both the exon and flanking introns, especially the intermediate intron. The strongly-correlated pairs also show a significant increase in the ORF preservation in a combinatorial manner. Sequence conservation, together with ORF preservation, indicates that strongly-correlated pairs are under more regulatory control and tend to be functionally important. The multiple-genome comparison further revealed that exon pairs with different correlation patterns may undergo different evolutionary courses. Most LNK pairs are old. The two exons in a LNK pair are usually of similar age, that is, similarly old. On the contrary, most IND pairs emerged in the recent genome evolution. The two exons in an IND pair are more likely to be of different age. The younger ones are frequently recruited from repeat elements. ME pairs lies between IND and LNK. There are many old and functional ME pairs, which mainly originated from exon duplication, while there are also many newly emerged ME events which seem to be less functionally important. Like in the IND group, quite a portion of the new ME pairs originates from repeat elements.

The evolution of individual alternative exons has two different paths: some alternative exons might have originated as a result of relaxation of the splicing signal which originally supported only constitutive splicing [16], while some other alternative exons might come from the mutations that turn an intron segment or repeat element directly into an alternative exon [34,42,43]. In the formation of an adjacent cassette exon pair, we speculate there are similar paths. The LNK exons in our analysis bear high resemblance to constitutive exons, in sequence conservation, high inclusion level and low selection pressure of frame preservation. The LNK exons were probably originally constitutive exons. With the weakening of the splicing signal and/or exon interaction effect, adjacent constitutive pairs can change into a LNK pair. The relative small difference of the exon ages in a pair also supports this hypothesis. On the other hand, there are frequently exon birth events during evolution. The new born exon may affect its neighboring exon and results in a correlated adjacent pair. Many exons in ME and IND are new and the two exons in a pair are often of different ages, so ME and IND pairs may often originate in this way. The exon ages were derived from multiple sequence comparison. With the accumulation of multiple genome alignment data (e.g. 28 vertebrate genomes alignment on UCSC bioinformatics site), we can determine the age to a finer scale. Then more delicate analysis (e.g. the continuous variable approach as in [44]) on the finer scale can tell us more about what happened during evolution.

We presented the primary regulatory and evolutionary properties for the differently interacting adjacent pairs. However, the splicing mechanism generating these interaction patterns is largely unknown. The IND pairs can be explained as independent control of each individual exon in a pair, but we do not know why the two exons in a ME pair are incapable of being spliced to each other or why the exons in a LNK pair are always included/excluded simultaneously. The possible mechanisms generating the different correlation patterns can be divided into "direct" and "indirect" interactions between the two exons in a pair. In direct interactions, the successful splicing of one exon promotes/prevents the splicing of the other one. Steric interference is one such direct interaction and possibly accounts for a portion of the ME cases. Spliceosome incompatibility [30] is another known direct interaction resulting in mutually exclusive splicing, though there is no evidence that it plays a role in our ME category (No "AT-AC" canonical splicing signal for U11/U12 spliceosome is observed in any ME case). These two known direct interactions, steric interference and spliceosome incompatibility, cannot explain all the cases we observed in the data.

An indirect interaction is when two exons in a pair are regulated independently by some other factors but the pooled observation generates spurious correlation. For example, tissue specificity could be such a factor. Suppose an adjacent pair is expressed in two tissues. The two exons are regulated in an independent but tissue-specific manner. In each tissue, the two exons are not correlated. When pooled together, the data shows strong negative correlation (Table 4). In this case, the cause of observed correlation is not the interaction between two adjacent exons but a controlling factor, tissue-specific regulation. The current amount of data cannot support a comprehensive analysis on the impact of tissue-specific regulation. EST numbers in different tissues are of high variance, which imposed further difficulties in this analysis. As a very primary step, we checked the role of tissue-specific regulation by Mantel-Haenszel test on a few tissues with relative large amount of data. The result indicates that most exon pairs, which are significantly correlated in pooled data in the term of Fisher exact test, remains significantly correlated in the term of Mantel-Haenszel test. On the other hand, a small proportion of pairs lost their Mantel-Haenszel significance, indicating the role of tissue-specific regulation on the formation of the observed correlation (See supplementary for detailed analysis and result).

**Table 4:** correlated pair by tissue-specific regulation

	**Tissue M**		**Tissue N**		**Pooled**	
	
exons	+D	-D	+D	-D	+D	-D
+U	9	81	9	1	18	82
-U	1	9	81	9	82	18

* +/- means the upstream (U) or downstream (D) exon in a pair is included/excluded in the transcript. In tissue M, the inclusion levels are 0.9 for U and 0.1 for D. In tissue N, the levels in 0.1 for U and 0.9 for D. The correlation coefficients between U and D are 0 in both M and N. But in pooled observation, the data show a strong negative correlation (-0.64). The cause of correlation in pooled data is not by the direct interaction between the two exons but the tissue-specific regulation.

We further tested an alternative, indirect hypothesis of the tissue-specific regulation: whether the genes hosting LNK and ME cases are expressed on average in a lower number of tissues. If true, this will give additional hints as to whether the strongly correlated pairs are especially regulated. However, we did not observe a significant difference in the tissue-specific expression of host genes (two approaches, based on EST and microarray data, see supplementary for details). This may be due to the fact that different subsets of genes are regulated in a tissue-specific manner at the transcriptional and alternative splicing levels [24,45]. The extraordinary conservation in flanking introns indicates the existence of regulatory motifs, but more comprehensive analysis and experimental verification should be carried out to explore the interaction mechanism, both direct and indirect interactions.

The formation of the exon correlation is complex and comes from various sources. Some correlated pairs may arise randomly. For example, most LNK pairs are in major form and some low abundance isoforms may be splicing artifacts. Despite various sources of the correlation, the differences between correlated and independent pairs reflect significant deviation from a random result, indicating the effect of natural selection. The next step will be to investigate the functions of these correlated pairs. Exon interaction can expand the protein encoding and regulation potential for transcripts. For example, it's interesting to explore the functions of the mutually exclusive pairs. Many ME pairs originate from exon duplication and the two ME exons are highly similar. Subtle changes in the sequence may play an essential role in the protein function, like a change in the catalytic site of an enzyme. Such analyses may reveal important results. Using Gene Ontology annotation[46], we performed functional enrichment analysis with GeneMerge [47]. But no significant enrichment was observed. Maybe the exon interaction is a prevalent phenomenon in transcripts and not enriched in a certain pathway or biological process.

There are several limitations in our work. First, as mentioned previously, we pooled all the ESTs from different libraries and can not distinguish the effect of tissue-specific control. This is due to a limited amount of data. A recent microarray analysis explored tissue-specific cassette exons and the exon correlation across more than 20 tissues and cell lines in mouse [24]. The results indicate many tissue-specific exon and correlated pairs are enriched in the brain. It's necessary to compare and combine the data from different sources in the future to investigate the correlation mechanism. The second limitation is that we studied only the interaction between adjacent cassette exons but not distant exons. Focusing on adjacent exons reflects a trade-off between an exhaustive description of the exon correlation and limited data coverage. Our analysis is mainly based on EST/cDNA sequences, which are usually local fragments of full-length gene transcripts, so correlation between distant cassette exons is out of the scope of this study. Full length sequence analysis by Emerick et al. discovered that non-adjacent exons interacted with one another in the CACNAIG gene [23]. The two correlated cassette exons in the two sequential EDI regions are also not adjacent in Fededa's experiments [21]. Nevertheless, since RNA splicing is coupled with transcription and splicing of exons is largely sequential from upstream to downstream [48,49], it could be expected that the adjacent exons are more likely to interact with each other than a distant pair. Our observation that the distances between two exons in strongly-correlated pairs are shorter than in loosely-correlated pairs supports this hypothesis. Our investigation of correlation between adjacent pairs showed only a small portion of the whole picture of exon interactions. With the accumulation of full length data and progress of new technology, like single molecule profiling [50], it will be possible to further investigate the complex interaction among exons in a gene, which will eventually provide us a fine picture and deeper understanding of alternative splicing and its regulation.

## Conclusion

We presented a genome-wide study of exon interactions in alternative splicing by recruiting the adjacent cassette exon pairs as a model. The adjacent cassette exon pairs show distinct interaction patterns, thus can be categorized into different groups. Compared with the loosely-correlated group, the strongly-correlated groups are more conserved and contain a higher proportion of pairs with reading frame preserved in a combinatorial manner. Additionally, the positively-correlated pairs bear strong resemblance to constitutive exons, which suggests that they may evolve from ancient constitutive exons. The negatively and weakly correlated pairs are more likely to contain newly emerging exons.

As the tissue-specific expression of a cassette exon indicates the possible function of this alternative splicing event, the specific exon interaction between alternative exons also shows they are under delicate splicing control and tend to be functionally important. But we know little about the mechanism of the observed interaction and its function, which represents the future direction of this study.

## Methods

### Calculation of exon correlation coefficients

The correlation coefficient was employed for its simplicity to describe the interaction of exons in an adjacent pair. Collecting all the ESTs covering this pair, the correlation coefficient is then calculated as

$$r=cor(u,d)=\frac{\sum_{i=1}^{n}\left(u_{i}-\bar{u}\right)\left(d_{i}-\bar{d}\right)}{\sqrt{\left(\sum_{i=1}^{n}{\left(u_{i}-\bar{u}\right)}^{2}\right)\left(\sum_{i=1}^{n}{\left(d_{i}-\bar{d}\right)}^{2}\right)}},$$

where $\bar{u}$, $\bar{d}$ are the means of *u *and *d*, respectively and *n *is the number of ESTs covering this region (Figure 1). Correlation coefficients always fall in the range [-1, +1]. A value of *r *= -1 means the two exons in a pair are mutually exclusive; meaning there is one and only one exon in each transcript. A value of *r = 1 *means the upstream and downstream exons are linked; meaning the two are simultaneously included or excluded. A value of *r *≈ 0 indicates the two exons are included or excluded in an independent manner.

### Adjacent cassette exon pair data set

Our data set of adjacent cassette exons was extracted from the Altsplice database of EBI (release2, April 2005)[25]. The records are predicted by an EST-based computational pipeline. The initial data for Altsplice was 898,295 high-quality EST-genome alignments in human and 837,329 alignments in mouse. Alternative splicing events in Altsplice are delineated as inconsistency between distinct splice patterns (EST-genome alignment). Thus an adjacent cassette exon pair will be identified in Altsplice data as either a mutually exclusive splicing (inconsistency between transcript structures 2 and 3, Figure 1) or an exon skipping event of two exons (inconsistency between transcript structure 1 and 4, Figure 1). We extracted all the data of these two alternative splicing types and the corresponding EST alignments covering this region deposited in Altsplice. This strategy generated the 4326 adjacent cassette exon pairs in human. The same pipeline was performed on Altsplice mouse data (release2, April 2005) and resulted in 1905 pairs. In several places, properties of individual cassette exons serve as the background when discussing adjacent pairs. These data are directly calculated from Altsplice annotation.

### Determination of the CDS and UTR location for exon pairs

We downloaded the knownGene annotations of human and mouse from the UCSC server [33] (knownGene.txt.gz for hg17 and mm5). The exon pairs are all mapped to the corresponding human/mouse genome. If the two exons in a pair were both located in a CDS region in knownGene annotation, this pair was treated as a CDS pair. Otherwise, this pair is an UTR pair.

### Sequence conservation curve of cassette exon pair and flanking introns

The genome alignments between human and mouse comes from the University of California, Santa Cruz Genome Bioinformatics Site. We downloaded the human-mouse alignments (hg17 vs mm5, [51]) for the human conservation curve in Figure 3 and mouse-human alignments for the mouse curve in Figure 4 (mm5 vs hg17, [51]). For the human-referenced percent identity curve, the insertions in the mouse genome were discarded and the gaps in the mouse are treated as mismatch. The procedure is similar for mouse-referenced curve. The curves show the average percent identity of the alignment in a 9-base sliding window.

### Conservation of cassette exon pair events

We took a different approach to explore the conserved cassette-exon pair event, that is, a cassette-exon pair in one species is also a cassette-exon pair in the other species. For a cassette-exon pair in human, we first found the mouse homolog gene of the human gene hosting this pair, via the BioMart [52]. If the mouse homolog also contains a cassette-exon pair (one of the 1905 pairs in our analysis), we aligned the two exons of human pair with the exons of mouse pair with Blast. The pair where both exons were conserved in mouse pair (E-value < 0.001) were categorized as a conserved pair. The same procedure was applied to the mouse data.

### Exon duplication

The homology between two exons in a pair was identified using the bl2seq implementation of TBLASTN. If the two exons showed significant homology, these two exons were considered as originating from exon duplication. In human and mouse, significance was set at E-values of 0.001, which is the same in Letunic's work [41].

### Overlapping with repeats

We used RepeatMasker[53] to identify interspersed repeat elements. The type and location of repeat elements are determined by parsing the ".out" output file. We used the "-s" parameter in the command line, which is the most sensitive setting when running RepeatMasker. If an exon shares at least one nucleotide with any repeat sequences, we thought it overlaps with repeat elements. An exon pair is overlapping with a repeat if it has one exon overlapping with any repeats.

### Evolutionary ages of exons

The evolutionary age of an exon is determined by the most distant species its ortholog can be found in. The rationale is that a human exon, whose ortholog is present in a given organism, must have been "born" before the divergence between human and that organism. This procedure is similar to the work of Zhang et al. [34]. We checked the orthologs of human exons in chimpanzee, dog, mouse, rat, chicken, zebrafish, and fugu genomes. (The choice of genomes is adopted from UCSC multiple alignments. See following.) Note that we did not require the orthologs in distant species to exist in intermediate lineages. For example, a human-fugu ortholog may be absent in the intermediate dog, mouse and chicken genomes. If an exon is human-specific (It's found only in the human genome), then the most distant ortholog is in human. By this way, each exon in our study can be associated with one of the eight vertebrate genomes. The eight species were further divided into 3 groups according to evolutionary history. The first group consists of human and Chimpanzee (divergence time: < 5 million years). The second group consist of dog, mouse and rat (divergence time: ~93 million years, [54]). The third group consists of all the rest of the species (chicken, zebrafish, and fugu, divergence time: > 310 million years). If the most distant ortholog of an exon falls into the first, second and third group, it will be classified as young, middle and old, respectively.

A similar procedure was performed on mouse exons. We checked the orthologs for mouse exons in rat, dog, human and chicken. The five organisms were also divided into three groups: 1) mouse and rat (divergence time: < 40 million); 2) dog and human (divergence time: 93 million); 3) chicken (divergence time: 310 million). According to the most distant ortholog, all the mouse exons are classified as young, middle and old.

The presence of ortholog exons in other genomes were determined by the multiple genome alignments from the University of California, Santa Cruz Genome Bioinformatics Site. We downloaded the human-referenced eight-genome alignment (hg17, multiz8way, [51]). The species in the multiple genome alignments are discussed above. The corresponding ortholog sequence of the second species was extracted according to the coordinates in the human exon if it is present in the UCSC alignment. The ortholog sequence was considered as a conversed exon only if the AG and GT di-nucleotides bordering this exon in the second species were conserved. This criteria has also been adopted by Zhang et al. [34]. For mouse exons, we also downloaded the mouse-referenced five-genome alignment (mm5, multiz5way, [51]) and determined their ortholog exons in other species.

## Authors' contributions

TP carried out the implementation of the methods, performed the analysis and mainly drafted the manuscript. CX helped in the experiment design and in drafting the manuscript. JB, XW and TL implemented part of the analysis and revised the manuscript. XZ helped in analyzing the results and revising the manuscript. YL conceived of the study and participated in its design and coordination. All authors have read and approved the final manuscript.

## Supplementary Material

Additional file 1 File with supplementary procedures, tables and figures Click here for file

Additional file 2 list of the adjacent alternative pairs in human and mouse, and the associated information. Click here for file
