# Supplementary material for: Functional importance of different patterns of correlation between adjacent cassette exons in human and mouse
Source: BMC Genomics. 2008 Apr 26;9:191. doi: 10.1186/1471-2164-9-191 (PMC2432081; doi:10.1186/1471-2164-9-191)
Supplement: Additional file 1 — File with supplementary procedures, tables and figures [file 1471-2164-9-191-S1.pdf]

Different patterns and functional importance of correlation between adjacent alternative exons in human and mouse

Tao Peng, Chenghai Xue, Jianning Bi, Tingting Li, Xiaowo Wang, Xuegong Zhang, Yanda Li§

MOE key laboratory of bioinformatics and Bioinformatics Division, TNLIST / Department of Automation, Tsinghua University, Beijing 100084, China

§Corresponding author

Re-sampling and permutation procedures

We re-sampled and permutated the EST evidence to make sure of two things: 1) the bias toward positive values in the distribution of correlation coefficient is not an artifact of small, discrete value of EST numbers; 2) the peaks on -1 and +1 in the distribution are not artifacts of biased distribution of correlation coefficients. These procedures were applied to human.

To address the first question, a re-sampling procedure was employed. First we constructed 3003 hypothetical pairs (the final number of human adjacent alternative pairs in our analysis) and the number of ESTs covering each pair was re-sampled from the true distribution of EST number (Figure S1). Then, we re-sampled the exon inclusion levels for upstream and downstream exons in each pair (Figure S4). By assuming that two exons in a pair are independent, the probability of the four types of ESTs (Figure 1) can be determined by taking the product of the marginal distributions. We sampled the ESTs from this joint distribution. Thus, for each hypothetical pair, a correlation coefficient can be obtained and the 3003 new correlation coefficients result in a re-sampled distribution. Figure S2A is an example of such a distribution. It is zero-centered, so the bias towards positive values is an intrinsic nature of the observed distribution of correlation

coefficients (Figure 2) and not an artifact. In the re-sampled distribution, we observed a small portion of data around -1. To compare it with the portion in the observed distribution, we performed a 100000 times re-sampling procedure and drew the distribution of proportions (# of pairs with $r < -0.7$ / # of all pairs, cutoff -0.7 is determined in the text) of all the re-samplings. The p value for observed portion (60/3003 = 0.002) is far less than 1e-5 (Figure S2C).

To address the second question, that the multimodality in the observed distribution (Figure 2A) is not an artifact of biased distribution of correlation coefficients, a permutation procedure was employed. We pooled all the ESTs covering the 3003 pairs together and randomly assigned them to the 3003 sets of original size. In each set, the same number of random ESTs and each pair get a new correlation coefficient. Figure S2B shows an example of the permuted distribution of correlation coefficients. It's a Gaussian-like distribution centered at 0.35, which captures the positive correlation in data but shows no multimodality. There is only a tiny peak at +1. To quantitatively describe this problem, we performed the permutation 100000 times and calculated the proportion of pairs with $r>0.7$ (the cutoff is the one used in the text to characterize the linked pairs.) for each permutation. The distribution of proportions of all permutations and the true value are drawn in Figure S2D. The P value for observed value is far less than 1e-5.

Fisher exact test for the cassette exon correlation

The correlation coefficient employed in our study is a straightforward but not strict metric to estimate the interaction between cassette exon pairs. The statistical test services as a more strict approach to verify our results. There are four possible isoforms for an exon pair. The numbers of each isoform can be summarized into a 2x2 contingency table. Fisher exact test is a proper approach for examining the correlation between the inclusion/exclusion statuses of these two exons.

We calculated the p-values for the cassette exon pairs in different groups (Table S5). The P-values of the pairs are consistent with the Pearson correlation coefficients: quite a proportion of pairs in

LNK and ME groups have a significant correlation in the term of Fisher exact test, while few pairs in IND group is significantly correlated. Primary analysis shows that the conclusions in manuscript (intermediate intron length, frame shift preservation, etc) remain the same when taking p-values as the grouping criteria (data not shown). The Fisher exact tests on all the cassette exon pairs form a multiple comparison problem in statistics. We did FDR (False Discovery Rate) correction for these tests. This more strict approach further reduced the number of pairs in each group, but the consistence with correlation coefficient remains: Almost all the significant correlated pairs in term of FDR belong to the ME or LNK group.

The P-values for each pair, together with the FDR (False Discovery Rate) correction result, are listed in additional file 2 for readers who concern high confident pairs. The Fisher p-value and FDR provide more strict results for case study and future experiment verification.

Calculation of splice site score

The splice site server of the Gil Ast lab was adopted to calculate the strength of each splice site [1] (http://ast.bioinfo.tau.ac.il/SpliceSiteFrame.htm). The server takes 9nt (-3, +6) to calculate the donor site score and 15nt (-14, +1) for acceptor site. The algorithm considers both position weight and mutual relationships among different positions. It deals with human and mouse splice sites separately.

Prediction of branch site and PPT

The tool to predict the branch site and Poly- pyrimidine tract (PPT) is also from the Gil Ast lab web service [2] (http://ast.bioinfo.tau.ac.il/BranchSite.htm). The algorithm locates both the BS and the PPT together by searching known combination of BS and PPT. The PPT borders are determined by a heuristic method based on experimental data. The algorithms dealing with human and mouse sequences are the same.

Tissue specificity of the correlated exon pairs

As in the Table 4 in manuscript, the observed correlation between adjacent cassette exons may be a spurious correlation caused by tissue-specific regulation. We performed Mantel-Haenszel test with the tissue source as stratifying variable, to give a primary estimate of the impact of tissue regulation on exon correlation.

We first mapped all the libraries of the EST to certain tissue by an automatic pipeline with manual correction, according to the annotation in the unified library database at NCBI (ftp://ftp.ncbi.nlm.nih.gov/repository/UniLib). This pipeline is similar with Xu's work [3]. 712387 human ESTs and 584114 mouse ESTs are categorized into 56 and 55 tissues, respectively. The EST counts in tissues are dramatically different, which makes it difficult to perform a comprehensive analysis across all tissues. For simplicity, we considered only brain, liver and eye, which have relative abundant EST data in both human and mouse. The EST counts for brain, liver and eye in human are 106363, 41100 and 39174, respectively. The EST counts in mouse are 122705, 32278 and 53708.

For a cassette exon pair, the ESTs from one tissue can be summarized to a 2x2 contingency table. Then, data from two tissues forms a 2x2x2 table, which can be checked by Mantel-Haenszel test. On the other hand, we can pool the data from two tissues together, construct a 2x2 table as one tissue and test this table by Fisher exact test. The p-values from Mantel-Haenszel test and Fisher exact test can give us a more detailed picture about the correlation mechanism of the cassette exon pair.

We repeated the analysis on every two tissues (brain-liver, liver-eye and eye-brain). The results are consistent (Table S4): most exon-pairs, which are significantly correlated in the term of Fisher exact test, remains significantly correlated in the term of Mantel-Haenszel test. A small proportion of pairs lost the significance when taking the tissue source as a stratifying variable in Mantel-Haenszel test, indicating a role of tissue-specific regulation in these pairs. However, most of the correlated exon pairs showed a direct interaction in a single tissue.

Tissue specificity of the host genes

The tissue-specific regulation of alternative splicing can be imposed on two levels [4]: 1) the gene level, which means that the gene hosting a certain alternative splicing event is tissue-specifically expressed, and 2) exon level, in which the host genes show no tissue specificity but the alternative exons are differently included / excluded in different tissues. Compared with the gene level, the exon level is more direct evidence that an alternative splicing event is regulated. In the last part, we analyzed the specificity on exon level in a few tissues. Here, we further tested the tissue-specificity on the gene level. If the genes carrying LNK and ME cases are expressed on average in a smaller number of tissues, this is an additional hint that LNK and ME events are under special regulation. We took two independent approaches to test this hypothesis. The first is based on the EST data we have; the second is based on microarray data.

In last part, the ESTs have been categorized into 56 and 55 tissues in human and mouse, respectively. Each human cassette pair was then associated with a 56-dimension vector, recording the number of supporting ESTs in all tissues. When normalized by the sum of vector elements (total number of ESTs covering this pair), the vector can be treated as a probability distribution, which is an estimator of the true probability distribution of tissues in which this pair is expressed. A large number of ESTs in a tissue means a large probability of gene expression in this tissue. The Shannon entropy of random variable was taken to test whether the genes carrying strongly-correlated pairs (LNK and ME) are on average expressed in fewer tissues. If a gene is uniformly expressed in many tissues, the EST hits among tissues are roughly equal. Then the probability of gene expression across tissues is evenly distributed. The Shannon entropy grows largest for random variables with an even distribution. On the other hand, if the EST is observed in only one tissue, the random variable for this gene becomes a deterministic variable and the Shannon entropy is zero. So, a larger entropy is a sign that the gene is expressed in limited tissues. The distribution of entropy for ME, IND and LNK groups in human is shown in Figure S6A. We did not observe any significant difference among groups. We obtained similar results for the mouse (Figure S6C).

The total numbers of all ESTs in different tissues is heavily biased by the EST sampling procedure. For example, the expression of a gene with ESTs count enriched in brain may not enrich in brain

in vivo. The great amount of EST in brain is just because many more ESTs are sampled from the brain than any other tissue. To relieve this limitation, we determined the background tissue distribution of all ESTs in our analysis. Taking the background distribution as prior and the observation distribution as posterior, we calculated the relative entropy (also called discriminant information) for each gene. The relative entropy can partly correct the bias in EST count. For example, a gene, whose ESTs are enriched in heart, is more specific than a gene with ESTs enriched in brain, because there are a lot more ESTs in the brain than the heart. The distributions of relative entropy for different groups are shown in Figure S6B and Figure S6D for human and mouse, respectively. However, there is also no significant difference in relative entropy distributions.

Powerful statistical method can weaken the impact of EST bias but not eliminate it. To make the results more confident, we employed an independent microarray dataset [5] for the tissue-specific expression analysis. The dataset contains expression data from across 79 tissues in human and 61 tissues in mouse. Microarray data don’t have the tissue biases that EST data have. There have been extensive works about finding tissue-specific genes with microarray data. We mapped the genes in ME, IND and LNK groups to array probe sets and found those which were tissue-specifically expressed by the standard z-score method [6]. The z-score method is simple and straightforward. The gene expression data across different tissues was normalized and z-score for each tissue was calculated. If the z-score for a tissue expression value exceeded certain threshold, that is, the expression value deviates greatly from the mean, the gene was recorded as specifically expressed in this tissue. Thus, the proportion of genes being specific in at least one tissue can represent the tissue-specificity of that group of genes. Those genes that are uniformly expressed in many tissues will unlikely be specific in certain tissue. The proportions of gene being specific in at least one tissue are listed in Table S4. In different groups, the proportions are similar.

Both of the two approaches, entropy on EST and z-score on microarray data, show that there are no significant differences in the tissue-specific expression of the genes hosting ME, IND and LNK events. This negative result may reflect the fact that different subsets of genes are regulated in a tissue-specific manner at the transcriptional and alternative splicing levels [7, 8].

ADDITIONAL TABLES

Table S1. Splice site scores

		Upstream Exon		Downstream Exon	
		acceptor	donor	acceptor	donor
human	ME	81.1 ± 7.8	79.6 ± 14.4	79.2 ± 8.6	79.5 ± 7.5
	IND	82.8 ± 7.4	81.1 ± 8.9	83.5 ± 7.2	80.8 ± 9.0
	LNK	82.7 ± 7.3	80.8 ± 9.8	82.5 ± 7.5	81.2 ± 9.3
mouse	ME	81.0 ± 8.2	86.3 ± 9.1	83.2 ± 8.6	85.3 ± 9.6
	IND	82.5 ± 7.9	86.1 ± 8.5	83.7 ± 7.7	85.8 ± 8.4
	LNK	83.5 ± 7.4	86.2 ± 10.2	82.9 ± 7.0	86.9 ± 8.7

- In each cell are mean and standard deviation of splice site scores.
- In human, the scores for constitutive acceptor and donor sites are 83.8 ± 7.4, 82.5 ± 8.6, respectively.
- In mouse, the scores for constitutive acceptor and donor sites are 83.9 ± 7.5, 87.4 ± 8.8, respectively.
- The procedure to calculate splice score is in the supplementary section “Calculation of splice site score”.

Table S2. Lengths of branch site and PPT to splice site (in nt)

		Upstream Exon		Downstream Exon	
		Branch site	PPT*	Branch site	PPT
human	ME	33.4±7.6	21.9±7.2	34.8±6.8	22.8±6.9
	IND	34.1±6.7	22.7± 6.6	34.8±7.2	23.2±7.0
	LNK	33.9±6.6	22.5± 6.4	33.9± 6.8	22.5±6.7
mouse	ME	33.8±7.3	22.8±7.9	36.9±7.5	24.9± 7.79
	IND	34.7± 7.0	23.2± 6.7	34.6±6.6	23.2±6.5
	LNK	33.6±6.7	22.4±6.4	34.4± 6.8	22.9±6.6

- * PPT stands for “poly-pyrimidine tract”.
- In each cell are mean and standard deviation of sequence lengths (distance from branch site to splice site or the length of PPT).
- The procedure to predict the branch site and PPT is in the supplementary section “Prediction of branch site and PPT”.

Table S3. Number of correlated pairs by tissue-specific regulation

		size	P-value 0.05			MDR 0.05		
			**Fisher**	**MH**	**Overlap**	**Fisher**	**MH**	**overlap**
**human**	**brain-liver**	682	68	52	48	10	7	7
	**liver-eye**	351	23	16	16	3	0	0
	**eye-brain**	813	94	68	67	18	12	12
**mouse**	**brain-liver**	150	7	6	6	2	2	2
	**liver-eye**	74	4	4	4	1	1	1
	**eye-brain**	371	58	38	38	14	13	13

- **Size** means the number of exon pairs which have enough ESTs in both pooled data (>10) and each of the two tissues (>1), which is the minimum data requirement for Fisher and Mantel-Haenszel test.
- **Fisher** and **MH** columns contain the number of pairs which is significant at the level of 0.05. **Overlap** is the number of pairs in common subset of the two test.
- **MDR** contains the results after False Discovery Rate correction.

Table S4. Proportions of tissue-specific genes in different groups

		# pairs	# with microarray data	# tissue-specific	# relative specific
**human**	**ALL**	4326	3206	499 (15.6%)	532 (16.6%)
	**ME**	60	52	9 (17.3%)	7 (13.5%)
	**IND**	1137	870	104 (12.0%)	121 (13.9%)
	**LNK**	957	780	116 (14.9%)	125 (16.0%)
**mouse**	**ALL**	1905	1409	220 (15.6%)	129 (9.16%)
	**ME**	60	41	3 (7.32%)	3 (7.32%)
	**IND**	424	317	38 (12.0%)	33 (10.4%)
	**LNK**	507	385	62 (16.1%)	28 (7.27%)

- Tissue-specific means a gene is specific in at least one tissue (with z-score in that tissue above a predetermined threshold).
- Relative-specific means one z-score of the gene is among the top of all z-scores in that tissue. Relative specific is another criterion like tissue-specific.
- The criteria for human and mouse are not the same. For mouse expression data is of higher variance than human, the criteria are stricter in mouse.

Table S5. The P-values in different exon correlation groups

		All	*P>0.05*	*P<0.05*	*FDR~0.05*
Human	*ME*	60	6	54	41
	IND	1137	1134	3	1
	LNK	957	185	772	517
Mouse	*ALL*	60	8	52	41
	IND	424	421	3	0
	LNK	507	103	404	228

- ***All*** means the number of cassette exon pairs in each group
- ***P>0.05*** means the number of pairs that are insignificant at the level of 0.05.
- ***P<0.05*** means the number of pairs that are significant at the level of 0.05.
- ***FDR~0.05*** means the number of pairs that remains significant after False Discovery Rate correction at the level of 0.05.

ADDITIONAL FIGURE LEGENDS

Figure S1. Number of EST evidences per adjacent alternative exon pair

(A) The distribution of EST number per adjacent alternative exon pair in human is shown in the graph. The peak on the right represents the number of grouped pairs with > 300 ESTs evidences. The red dashed line corresponds to 10 ESTs. Those exon pairs with <= 10 ESTs are ignored in our analysis.

(B) This histogram is for data in mouse. All the marks are the same with those in (A).

Figure S2. Re-sampling and permutation results for correlation coefficients

(A) This figure shows an example of the re-sampled distribution of correlation coefficients. It is a zero-centered Gaussian-like distribution, indicating that the bias towards positive values in the distribution is intrinsic and not a random event. See supplementary text for the re-sampling procedure. (B) We also performed a permutation procedure to validate that the multimodality in the true distribution is not an artifact of the bias towards positive values. An example distribution after permutation is shown in this figure. It is a Gaussian-like distribution centered at 0.35. Though there is a little peak at +1, the multimodality in this distribution is far different from the

true one. (C) The x-axis in this figure is the proportion of pairs with $r<-0.7$ (# of pairs with $r<-0.7$ / # of all pairs) and the y-axis is the number of pairs in each bin. The re-sampling procedure was performed 100000 times and the distribution of the proportion is drawn in the figure. The observed proportion is indicated by the red arrow. (D) The x-axis is the proportion of pairs with $r>0.7$ (# of pairs with $r>0.7$ / # of all pairs). The permutation procedure was performed 100000 times and the distribution of the proportion is drawn. The observed proportion is indicated by the red arrow.

Figure S3. Inclusion level of individual cassette exon

The x-axis is the inclusion level (defined as the fraction of transcripts that include this pair rather than skipping it) for each individual cassette exon. The y-axis is the number of exons. The inclusion levels are strongly biased to both 0 and 1. The two isoforms in an alternative splicing event tend to be differently expressed. The distributions are similar in human and mouse.

Figure S4. Inclusion level of adjacent pairs

The x-axis is the inclusion level and the y-axis is the number of pairs (same with Figure S3). The distribution of adjacent alternative pairs is similar to that of individual cassette exons, but there are more exons that are included in a high level (towards 1). The distributions are similar in both human and mouse.

Figure S5. Permutation for the age differences between upstream and downstream exons

We pooled the age labels of exons and randomly assigned them to upstream and downstream exons. For each permutation, we calculated the fraction of pairs with a younger upstream exon. (Ny/(Ny+No), where Ny is the number of pairs with a younger upstream exon and the No is the number of pairs with an older upstream exon.) The distribution of the fractions of 100000 permutations for each group is drawn in the Figure. (A), (B), (C) are for human and (D), (E), (F) are for mouse. The observed fractions calculated from Table 3 are indicated as a red arrow in each

subfigure. The hypothesis that the upstream exon is younger than the downstream one is significant in IND and LNK groups in both human and mouse. But the difference is not significant in the ME group, probability due to the limited amount of data.

Figure S6. Distribution of the entropy measuring tissue specificity

(A) A pair is associated with an entropy value telling the tissue specificity of the host genes. The distribution of entropy values for each group in human are drawn in (A). From the top-down are ME, IND and LNK groups. (B) This figure is also for human, similar to figure (A). The values in distribution are relative entropy, rather than entropy (see text for details). (C) and (D) are mouse distribution of entropy and relative entropy, respectively. The arrangement of groups is the same as in (A) and (B). The pairs in ME are far fewer than IND and LNK groups, so the number of bins for ME is also fewer than the other two groups. We can not observe significant difference among groups in all of the four figures.

ADDITIONAL FIGURES

Figure S1

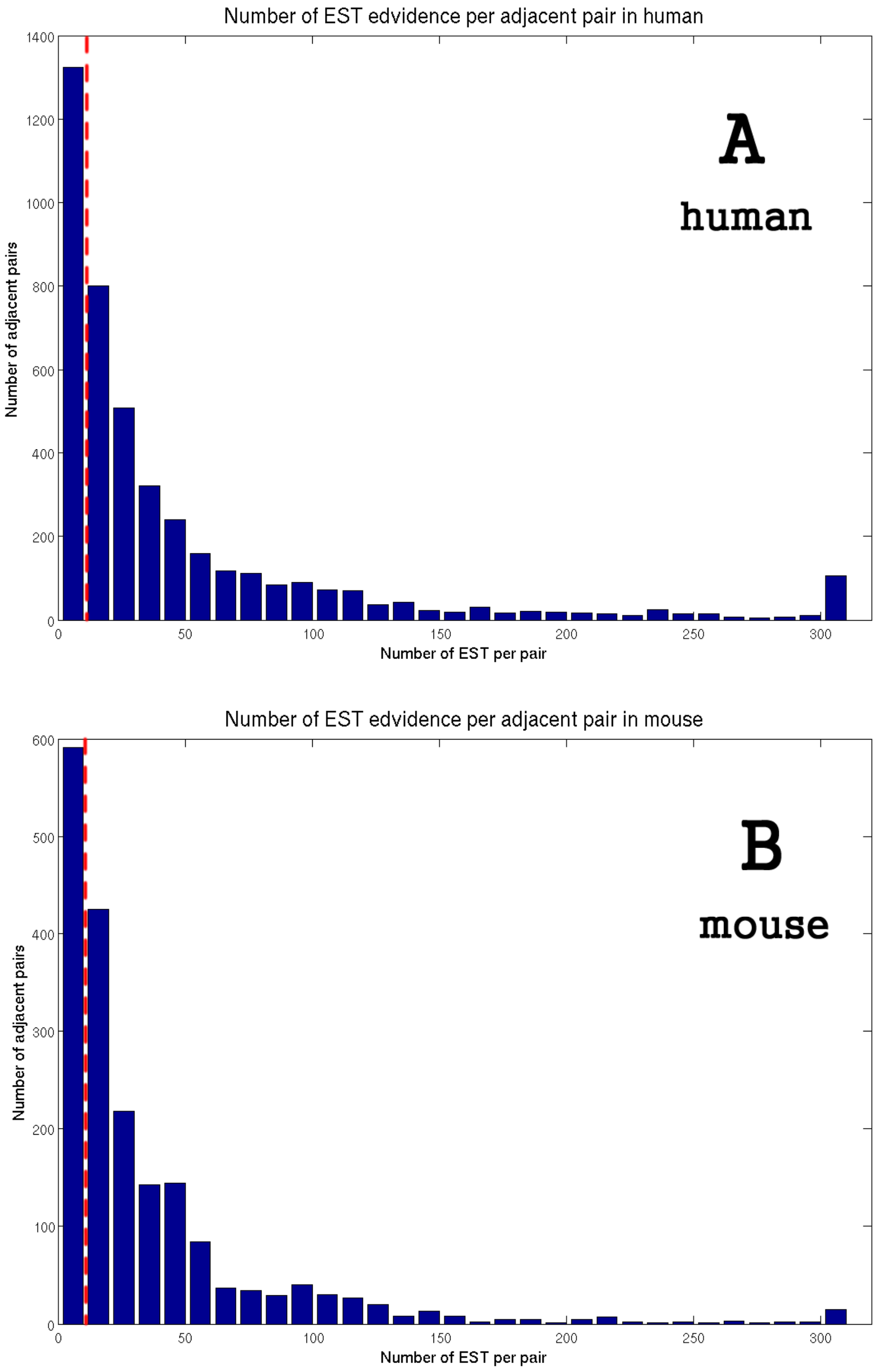

Figure S2

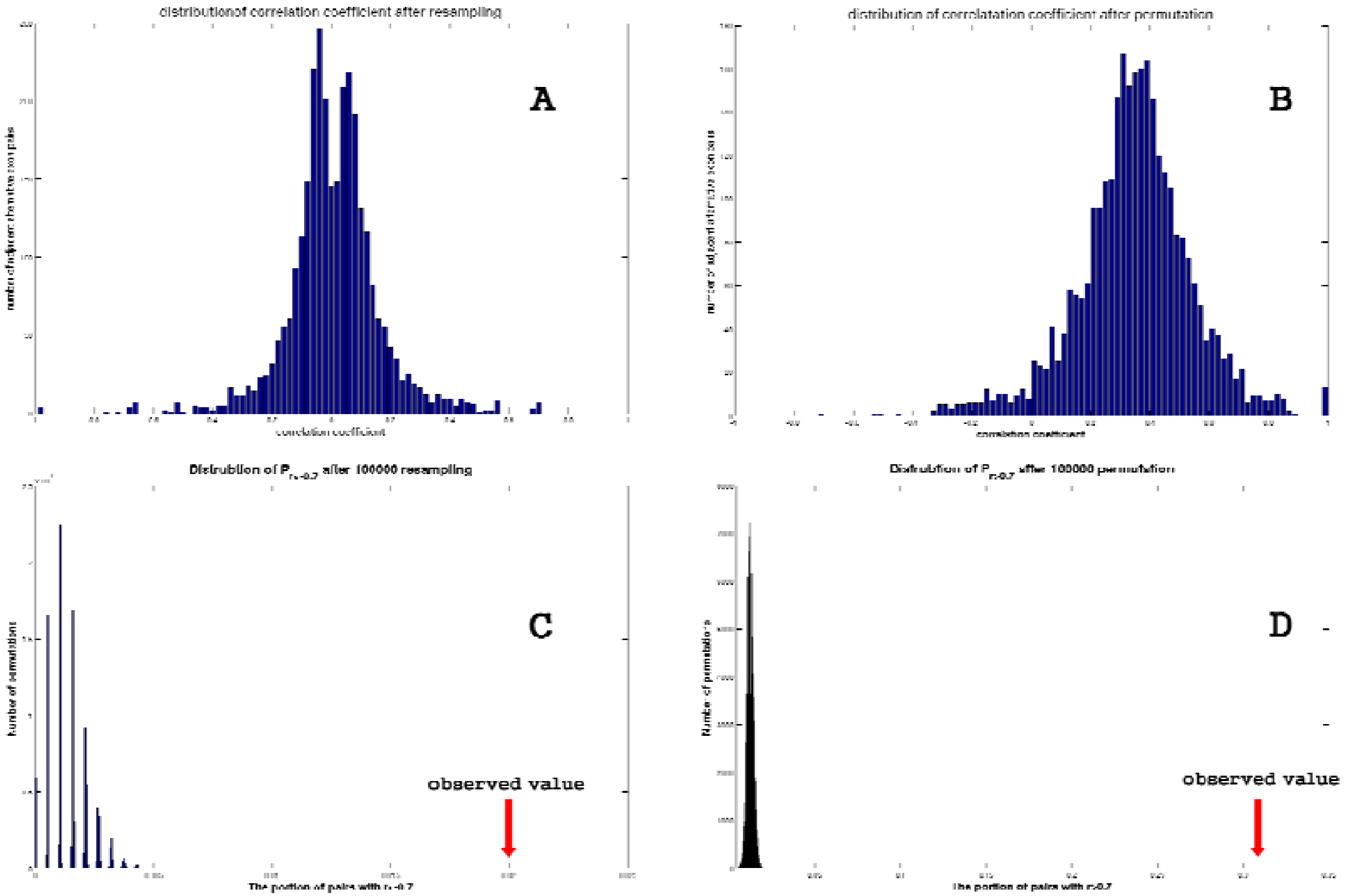

distributionof correlation coefficient after resampling
A
correlation coefficient
distribution of correlatation coefficient after permutation
B
C
observed value
D
observed value

Figure S3

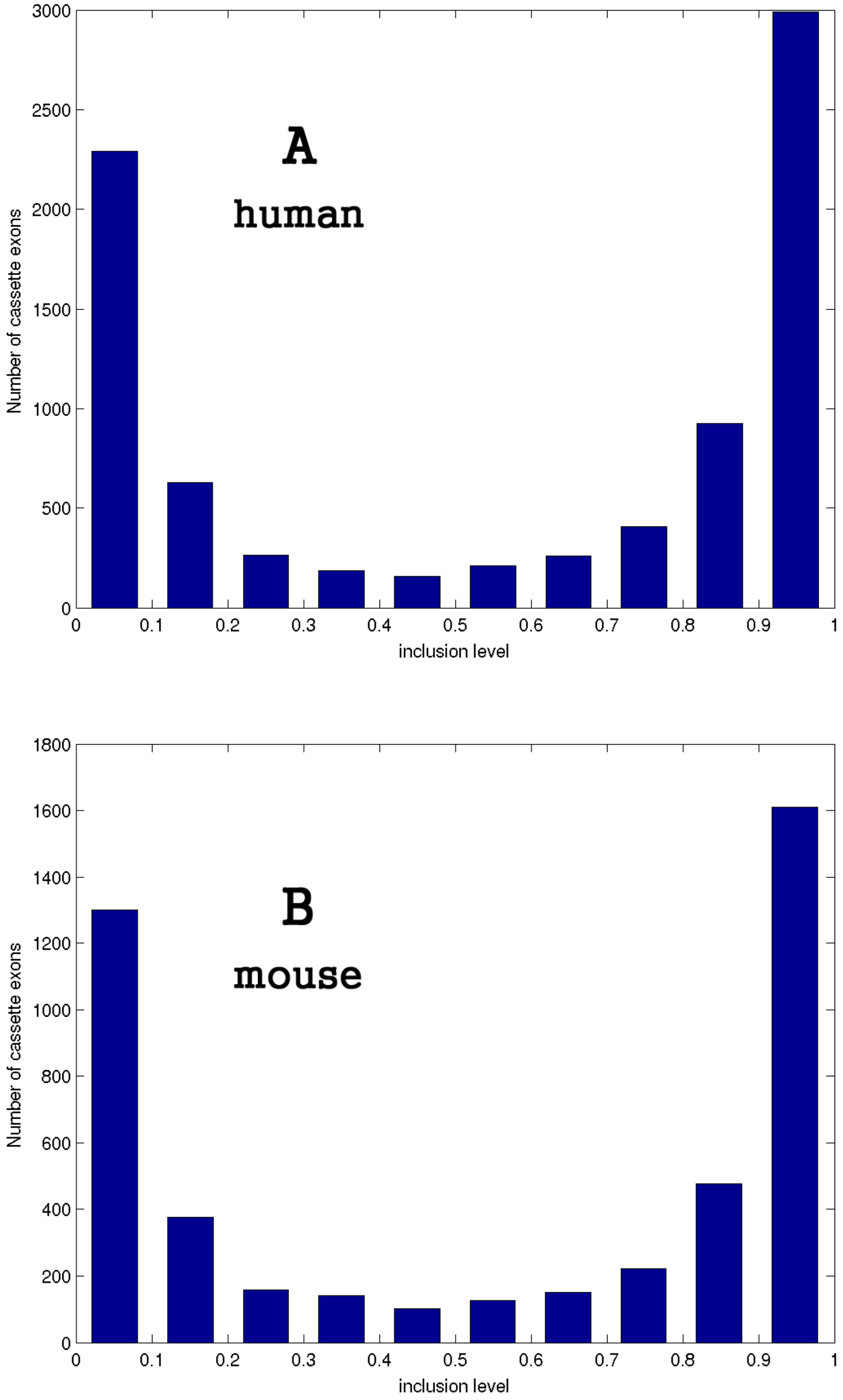
A
human
Number of cassette exons
3000
2500
2000
1500
1000
500
0
0
0.1
0.2
0.3
0.4
0.5
0.6
0.7
0.8
0.9
1
inclusion level
B
mouse
Number of cassette exons
1800
1600
1400
1200
1000
800
600
400
200
0
0
0.1
0.2
0.3
0.4
0.5
0.6
0.7
0.8
0.9
1
inclusion level

Figure S4

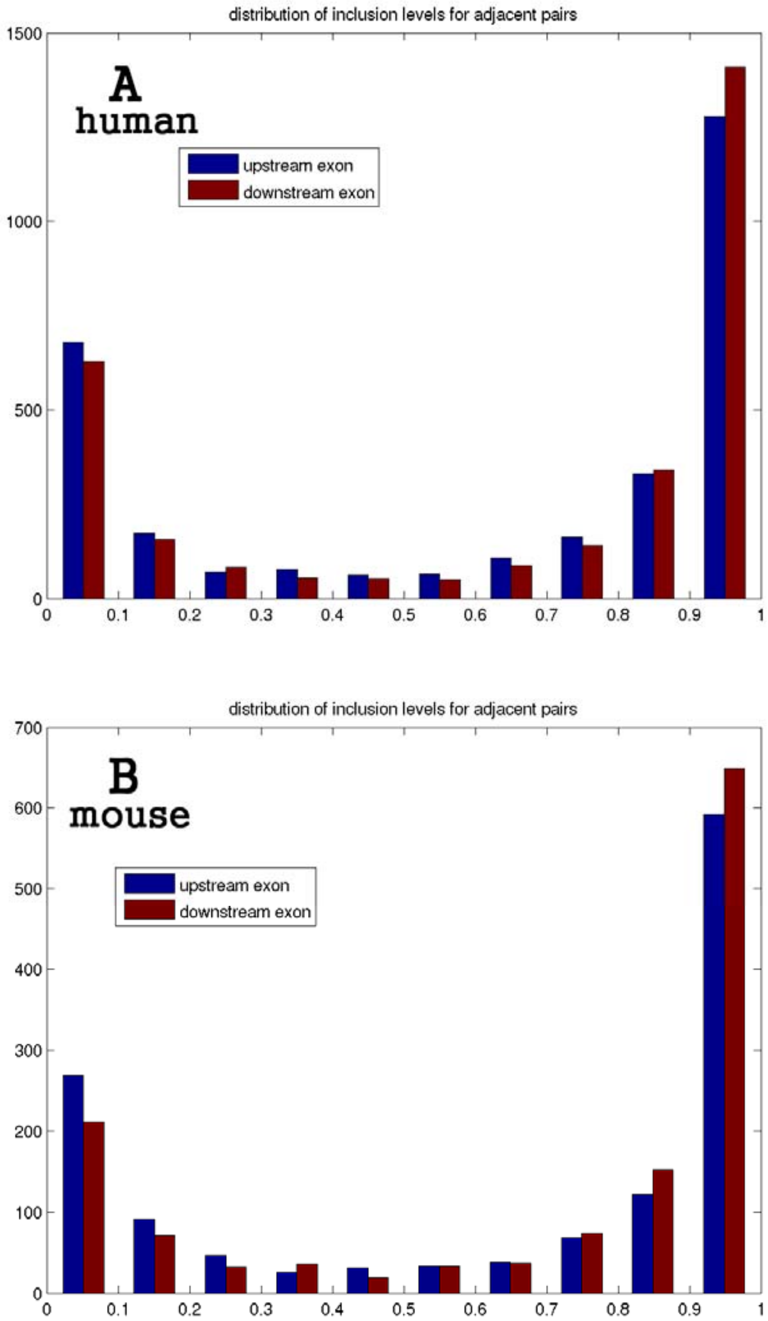
distribution of inclusion levels for adjacent pairs
A
human
upstream exon
downstream exon
1500
1000
500
0
0
0.1
0.2
0.3
0.4
0.5
0.6
0.7
0.8
0.9
1
distribution of inclusion levels for adjacent pairs
B
mouse
upstream exon
downstream exon
700
600
500
400
300
200
100
0
0
0.1
0.2
0.3
0.4
0.5
0.6
0.7
0.8
0.9
1

Figure S5

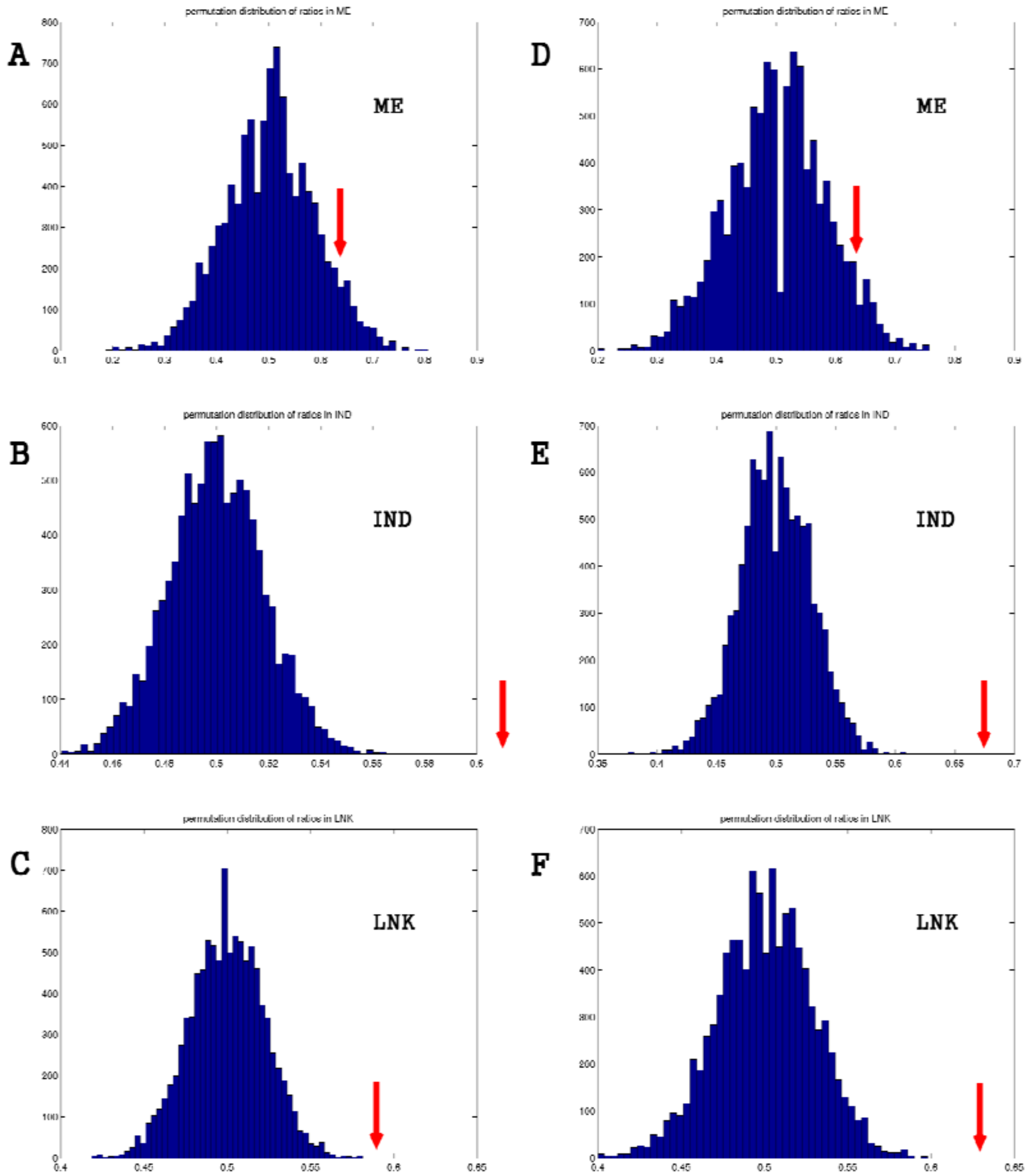
A
ME
permutation distribution of ratios in ME
D
ME
permutation distribution of ratios in ME
B
IND
permutation distribution of ratios in IND
E
IND
permutation distribution of ratios in IND
C
LNK
permutation distribution of ratios in LNK
F
LNK
permutation distribution of ratios in LNK

Figure S6

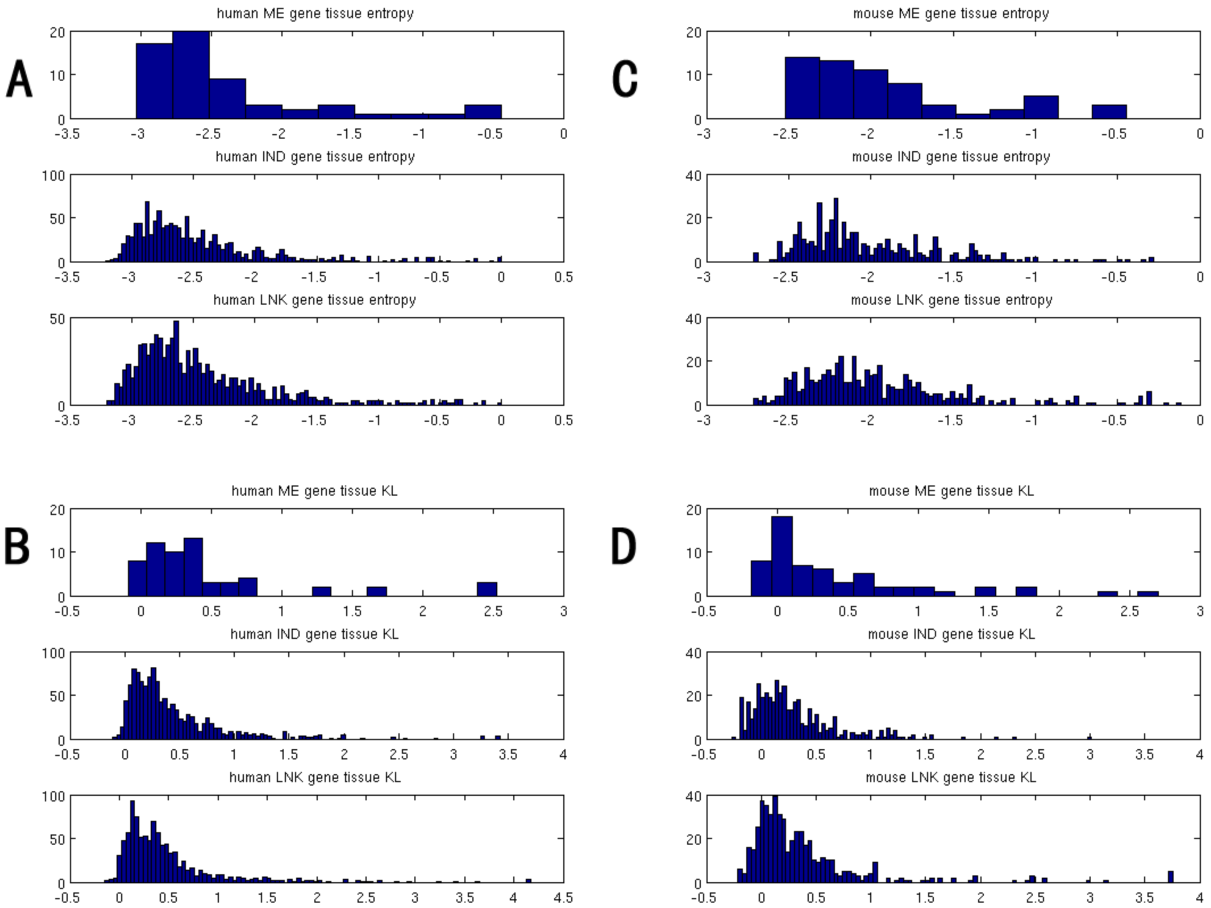
A
human ME gene tissue entropy
human IND gene tissue entropy
human LNK gene tissue entropy
B
human ME gene tissue KL
human IND gene tissue KL
human LNK gene tissue KL
C
mouse ME gene tissue entropy
mouse IND gene tissue entropy
mouse LNK gene tissue entropy
D
mouse ME gene tissue KL
mouse IND gene tissue KL
mouse LNK gene tissue KL

REFERENCES

1. Carmel, I., et al., *Comparative analysis detects dependencies among the 5 ' splice-site positions.* Rna-a Publication of the Rna Society, 2004. **10**(5): p. 828-840.
2. Kol, G., G. Lev-Maor, and G. Ast, *Human-mouse comparative analysis reveals that branch-site plasticity contributes to splicing regulation.* Human Molecular Genetics, 2005. **14**(11): p. 1559-1568.
3. Xu, Q., B. Modrek, and C. Lee, *Genome-wide detection of tissue-specific alternative splicing in the human transcriptome.* Nucleic Acids Research, 2002. **30**(17): p. 3754-66.
4. Blencowe, B.J., *Alternative splicing: New insights from global analyses.* Cell, 2006. **126**(1): p. 37-47.
5. Su, A.I., et al., *A gene atlas of the mouse and human protein-encoding transcriptomes.* Proceedings of the National Academy of Sciences of the United States of America, 2004. **101**(16): p. 6062-6067.
6. Smith, A.D., et al., *DNA motifs in human and mouse proximal promoters predict tissue-specific expression.* Proceedings of the National Academy of Sciences of the United States of America, 2006. **103**(16): p. 6275-6280.
7. Fagnani, M., et al., *Functional coordination of alternative splicing in the mammalian central nervous system.* Genome Biol, 2007. **8**(6): p. R108.
8. Pan, Q., et al., *Revealing global regulatory features of mammalian alternative splicing using a quantitative microarray platform.* Mol Cell, 2004. **16**(6): p. 929-41.
